# Aberrant expression of CPSF1 promotes head and neck squamous cell carcinoma via regulating alternative splicing

**DOI:** 10.1371/journal.pone.0233380

**Published:** 2020-05-21

**Authors:** Akihiro Sakai, Mizuo Ando, Takahito Fukusumi, Shuling Ren, Chao Liu, Jesse Qualliotine, Sunny Haft, Sayed Sadat, Yuki Saito, Theresa W. Guo, Guorong Xu, Roman Sasik, Kathleen M. Fisch, J. Silvio Gutkind, Elana J. Fertig, Alfredo A. Molinolo, Joseph A. Califano

**Affiliations:** 1 Moores Cancer Center, University of California San Diego, San Diego, California, United States of America; 2 Department of Otolaryngology, Head and Neck Surgery, Tokai University, Isehara, Japan; 3 Department of Otolaryngology, Head and Neck Surgery, Xiangya Hospital, Central South University, Changsha, Hunan, China; 4 Division of Otolaryngology, Head and Neck Surgery, Department of Surgery, University of California San Diego, San Diego, CA, United States of America; 5 Department of Otolaryngology, Head and Neck Surgery, Johns Hopkins Medical Institutions, Baltimore, Maryland, United States of America; 6 Center for Computational Biology and Bioinformatics, Department of Medicine, University of California San Diego, San Diego, CA, United States of America; 7 Department of Pharmacology, University of California San Diego, San Diego, CA, United States of America; 8 Division of Oncology Biostatistics and Bioinformatics, Sidney Kimmel Comprehensive Cancer Center, Department of Oncology, Johns Hopkins University, Baltimore, MD, United States of America; 9 Department of Pathology, University of California San Diego, San Diego, California, United States of America; Augusta University, UNITED STATES

## Abstract

Alternative mRNA splicing increases protein diversity, and alternative splicing events (ASEs) drive oncogenesis in multiple tumor types. However, the driving alterations that underlie the broad dysregulation of ASEs are incompletely defined. Using head and neck squamous cell carcinoma (HNSCC) as a model, we hypothesized that the genomic alteration of genes associated with the spliceosome may broadly induce ASEs across a broad range of target genes, driving an oncogenic phenotype. We identified 319 spliceosome genes and employed a discovery pipeline to identify 13 candidate spliceosome genes altered in HNSCC using The Cancer Genome Atlas (TCGA) HNSCC data. Phenotypic screens identified amplified and overexpressed CPSF1 as a target gene alteration that was validated in proliferation, colony formation, and apoptosis assays in cell line and xenograft systems as well as in primary HNSCC. We employed knockdown and overexpression assays followed by identification of ASEs regulated by CPSF1 overexpression to identify changes in ASEs, and the expression of these ASEs was validated using RNA from cell line models. Alterations in expression of spliceosome genes, including CPSF1, may contribute to HNSCC by mediating aberrant ASE expression.

## Introduction

Head and neck squamous cell carcinomas (HNSCC) constitute the sixth most common cancer type in the world [1]. The most important conventional risk factors for HNSCC are tobacco and excessive consumption of alcohol [2]. In addition to these factors, human papillomavirus (HPV) is recognized as an independent risk factor for oropharyngeal squamous cell carcinomas and cervical cancer [3]. The Cancer Genome Atlas (TCGA) study also reported differences in genetic alterations between HPV-negative and -positive cancers, underscoring that HPV-positive HNSCC are a distinct tumor type from HPV-negative HNSCC. Comprehensive examination of genomic alterations in HNSCC was reported by investigators from The Cancer Genome Atlas (TCGA) Research Network showed that many HNSCC have alterations in genes for growth factor receptors (EGFR, FGFR, IGFR, MET, ERBB2, and DDR2), signaling molecules (PIK3CA and HRAS), and regulation of genomic integrity and the cell cycle (TP53, CCND1). [4] These genes may play an important role in controlling cell growth and proliferation, and many therapies targeting these genes are available or in development, however, an improvement in survival rate has not yet been observed.

Alternative RNA splicing is a mechanism that generates multiple different mRNAs and produces multiple proteins and functions from a single gene. Approximately 92%–94% of human genes are considered to undergo alternative splicing [5]. Alternative splicing has been demonstrated to have a key role in cancer development, and data indicate that alternative splicing of key genes such as BCL2L1 [6], RON [7] and FOX2 [8] can drive a cancer phenotype. In fact, Karni et al. [9] reported that the dysregulated expression of SRSF1 could cause oncogenic transformation of cells. However, only a few studies have investigated alternative splicing in HNSCC [10, 11].

We defined an alternative splicing event phenotype and showed that alternative splicing could represent an important contributor to HPV-related oropharyngeal cancer[12]. We assumed that the oncogenesis driven by alternative splicing could be associated not only with HPV-positive tumors but also HPV-negative tumors. If a gene that regulates splicing is aberrant, the resulting change in splicing can cause a significant change in function. Thus, we hypothesized that a contributor to HNSCC development may be spliceosome gene mutations and/or overexpression, which could induce aberrant splicing in HNSCC.

We underwent a broad, genome wide approach to discover potential contributors of ASE in HNSCC, and screened components of the spliceosome using functional assays followed by additional validation, defining CPSF1 as a spliceosome gene with potential oncogenic contributor functions associated with broad gene splicing alterations.

## Materials and methods

### 1. Determination of the candidate genes from the spliceosome gene set

A list of annotated spliceosome genes based on gene ontology (GO) accessions was created. Using the QuickGO website (https://www.ebi.ac.uk/QuickGO/), the GO database was searched for the term “spliceosome” as well as for all of its descendent terms, and 319 spliceosome genes were identified. To investigate the relationship between the 319 genes and ASEs, the ASEs were calculated using the same method as previously described [12]. We defined ASEs as a splice variant with significantly higher outliers in tumors (46 HPV-positive oropharyngeal cancers) compared to normal samples (25 normal UPPP samples) defined as junction count normalized by gene expression defined by RSEM as previously reported. We used the multiple filters and outlier statistics by RNA sequence, 109 ASEs with significantly higher outliers in tumors were discovered.

A heatmap and hierarchical clustering of gene expression levels between the samples and the ASE number in the Johns Hopkins cohort was created using the function “heatmap.2” in the “gplots” package of R. The TCGA data from 279 head and neck cancer samples were then used to obtain an overview of the alterations (mutation, copy number variation (CNV), and expression (z-score threshold ± 2.0)) of the 319 spliceosome genes in HNSCC using cBioPortal (http://www.cbioportal.org/). From these results, the list was sorted by frequency of mutations, as well as frequency of copy number variation and expression alterations. The 319 genes were narrowed down to 13 candidate genes with alterations most consistent with potential oncogenic function for further analysis. 13 genes were selected based on high number of mutations and/or CNV, with accompanying expression alterations. Genes selected for analysis were based on a 1) mutation threshold of 3 or greater tumors 2) high frequency of upregulation and/or downregulation and 3) concordance of amplification with upregulation or deletion with downregulation, 3) consistent direction of amplification/upregulation or deletion/downregulation, 4) high total frequency of these alterations, and 5) consistency of these alterations with biologic activity reported in the literature.

### 2. Screening of candidate genes

#### 2.1 Cell line selection

For cell line selection, mRNA expression data from 22 head and neck cancer cell lines were analyzed to determine the cell line most appropriate for these experiments. Expression data from the OPC-22 panel were kindly provided by the Gutkind Laboratory [13] (S5 Table). Cell lines with the top five highest mRNA expression levels of a candidate gene were selected for each gene, and two cell lines for each gene were selected and used for this assay. The mutational status and copy number variations in each cell lines we used were summarized (S6 Table).

#### 2.2 Cell culture

The UD-SCC2 (RRID:CVCL_E325), 93VU147T (RRID:CVCL_L895), UPCI-SCC090 (RRID:CVCL_1899), UM-SCC17B (RRID:CVCL_7725), BHY　(RRID:CVCL_1086), Detroit562　(RRID:CVCL_1171) and UM-SCC11B　(RRID: CVCL_7716) cell lines were kindly provided by the Gutkind Laboratory at the University of California San Diego, Moores Cancer Center. These cell lines were authenticated previously [13]. The SCC-9　(RRID:CVCL_1685) cell line was obtained from the American Type Culture Collection (ATCC). The BICR22 (RRID:CVCL_2310) cell line was purchased from Sigma–Aldrich (Sigma–Aldrich, St. Louis, MO, USA). The SCC-9 and BICR22 cell line were authenticated by the manufacturer. After thawing, cells were usually cultured for no longer than 2–3 months. Routine Mycoplasma testing was performed by PCR. The BICR22 cell line was cultured in Dulbecco’s modified Eagle’s medium (DMEM, Sigma–Aldrich) supplemented with 10% fetal bovine serum (FBS, GIBCO, Carlsbad, CA, USA), 2 mM glutamine, 0.4 mg/ml hydrocortisone, and a penicillin (50 U/ml) and streptomycin (50 μg/ml) cocktail. SCC-9 was cultured in a 1:1 mixture of DMEM and Ham's F12 medium supplemented with 10% FBS, antibiotics, and 0.4 mg/ml hydrocortisone. The other cell lines were cultured in DMEM supplemented with 10% FBS and antibiotics. All cell lines were cultured under an atmosphere of 5% CO2 at 37°C.

#### 2.3 siRNA transfection

The siRNAs for the knockdown of candidate genes were purchased from Dharmacon. ON-TARGETplus SMARTpool was used for PRPF6 (L-012821-01), DBR1 (L-008290-00), PSIP1 (L-015209-00), SNRPN (L-011776-02), SRPK2 (L-004839-00), DHX9 (L-009950-00), TRA2B (L-007278-00), RSRC1 (L-020548-01), CPSF1 (L-020395-00), RBM4 (L-019588-00), HNRNPL (L-011293-01), YTHDC1 (L-015332-02), and CPSF7 (L-015842-02), and the scrambled ON-TARGETplus non-targeting siRNA pool (D-001810-10) was used as a control. The siRNAs for the validation of CPSF1 knockdown were purchased from Origene. The siRNA Oligo Duplex was used for CPSF1 (SR309262) and the Trilencer-27 Universal scrambled negative control siRNA duplex (SR30004) was used as a control. All cell lines were transfected using Lipofectamine RNAi MAX (Thermo Fisher Scientific) according to the manufacturer's instructions.

#### 2.4 Proliferation assays

Cells were plated in 96-well plates at 3,000–8,000 cells/well, with five replicates per experiment. Growth was measured on the day of transfection and at 24, 48, and 72 h after transfection using the Vita Blue Cell Viability Reagent (bimake.com, Houston, TX, USA). These assays were performed four times to confirm consistent proliferation effects.

#### 2.5 Screening of candidate genes by siRNA transfection

Specific siRNA for each gene were selected, and growth curves were compared between the si-control and si-target gene knockdown cells.

### 3. Identification of ASEs in the TCGA HNSCC cohort

ASEs in the TCGA HPV-positive and -negative cohorts were identified using the outlier analysis algorithm as previously described [12]. Briefly, RNA sequencing data from TCGA was realigned to obtain junction expression using the MapSplice2 pipeline. Gene expression data were determined using publicly available RSEM (RNA-Seq by Expectation-Maximization) data. Then, splice variant identification and outlier statistics were applied to these junction data from TCGA using the same R-code developed by Guo et al. [12] to identify significant splice variants within the HPV-positive and -negative TCGA tumors (407 HPV-negative tumors and 90 HPV-positive tumors) compared with 44 normal samples [14]. The total numbers of significant splice variants per sample (the number of ASEs) were calculated using these data.

### 4. Plasmids and stable transfections

The pLenti-C-CPSF1-mGFP-P2A-Puro and pLenti-C-mGFP-P2A-Puro empty vectors were purchased from OriGene (OriGene Technologies, Inc., Rockville, MD, USA). Lentiviral particles were prepared for CPSF1 and empty vector expression using 293T cells as the packaging cells. SCC090 and SCC17B cells were infected with viral supernatants for 24 h at 37°C in the presence of 8 μg/ml Polybrene (hexadimethrine bromide; Sigma). The transfected cells were selected using 1 μg/ml Puromycin (Invivogen, San Diego, CA, USA).

### 5. Inducible stable shRNA transfection

Inducible shRNA expression vectors (SMARTvector Inducible Human CPSF1 hEF1a-TurboGFP shRNA (V3SH11255-02EG91746, V3SH11252-226572495, V3SH11252-227528901, and V3SH11252-227628825) and SMARTvector Inducible Non-targeting hEF1a-TurboGFP (VSC11653) were purchased from Dharmacon (GE Healthcare Dharmacon, Inc., Chicago, IL, USA). Lentiviral particles were prepared for CPSF1 and non-targeting shRNA expression as described above. BICR22 and Detroit562 cells were infected with viral supernatants containing CPSF1 shRNA with Polybrene, followed by selection using 1 μg/ml Puromycin. After infection by virus, the cells were cultured in DMEM with 10% Tet-System-Approved FBS (Takara Bio USA, Inc., Mountain View, CA, USA), 2 mM glutamine, 0.4 mg/ml hydrocortisone, and antibiotics.

### 6. Colony formation assays

Approximately 3,000 cells were added to each well of a six-well culture plate, and each experiment was performed in triplicate. After 12 days of culture at 37°C, the cells were fixed and stained using a Differential Quik Stain Kit (Polysciences, Inc., Warrington, PA, USA). Visible colonies were manually counted under a microscope.

### 7. Apoptosis assays

Apoptosis was determined by cell staining using an Annexin V-FITC Apoptosis Detection Kit (Sigma–Aldrich, Inc., Saint Louis, MO, USA). Briefly, the cells were collected, washed twice with cold phosphate-buffered saline, and co-stained with Annexin V and propidium iodide according to the manufacturer’s protocol. Apoptotic cells were analyzed using a BD FACS Calibur (BD Biosciences Corporation, Franklin Lakes, NJ, USA). Data analysis was conducted using the FlowJo software.

### 8. Quantitative real-time PCR

Total RNA was extracted from the cell lines using the RNeasy plus mini kit (Qiagen, Hilden, Germany), and complementary DNA was synthesized using a high-capacity cDNA reverse transcription kit (Thermo Fisher Scientific, Waltham, MA, USA). The primers used for mRNA expression were obtained from TaqMan Gene Expression Assays (catalog number: #4331182, Thermo Fisher Scientific). The gene IDs were GAPDH: Hs02758991_g1 and CPSF1: Hs00273612_m1. The housekeeping gene GAPDH was used as an internal control. Quantitative reverse transcription-polymerase chain reaction (qRT-PCR) was performed using the Quant Studio 6 Flex Real-time PCR System (Thermo Fisher Scientific).

### 9. Western blot

Cells were lysed using ice-cold RIPA buffer containing 50 mM Tris at pH 7.4, 0.5% sodium deoxycholate, 150 mM sodium chloride, 2 mM EDTA, 0.1% SDS, 1% NP-40, 50 mM NaF, and protease and phosphatase inhibitor cocktails. The protein concentrations were measured using the Protein Assay Kit (DC^™^ Protein Assay, BIO-RAD). Equal amounts of denatured protein were loaded for Western blot assay using Mini-PROTEAN TGX gels (Bio-Rad). The following primary antibodies were used for analysis: CPSF1 (Abcam ab62598) and GAPDH (Santa Cruz SC25778). The blots were incubated overnight at 4°C. Horseradish peroxidase-conjugated goat anti-rabbit Ig (7074) was used as a secondary antibody. ECL (Pierce™ ECL Western Blotting Substrate, Thermo Scientific™) was used for Western blot development.

### 10. *In vivo* tumor growth assay

SCC090 cells transfected with CPSF1 or empty vector (2×10^6^cells/200 μL DMEM with matrigel) were injected subcutaneously into the flanks of female nude mice. Tumor volumes were measured twice a week and calculated using the following formula: Tumor Volume = (π × Width^2^ × Length)/6. Tumor size was observed over a 60-day period. The mice were euthanized using carbon dioxide gas for over 10 minutes. Tumor was removed and tumor weight was measured after sacrifice. Ten mice were used in each experiment. These experiments were performed in triplicate. Experiments were approved by the Institutional Animal Care and Use Committee of the University of California San Diego.

### 11. CPSF1 immunohistochemistry on tissue microarray

A tissue microarray containing 224 total tissue cores, including 22 cores of non-neoplastic squamous epithelium and 202 cores of head and neck squamous cell carcinoma representing a broad distribution of primary sites was obtained from the Johns Hopkins University Head and Neck Cancer Tissue Bank. Anti-CPSF1 antibody (ab81552) was purchased from Abcam (Cambridge, UK) and used to perform immunohistochemistry on the tissue microarray at a 1:600 dilution. The processed tissue microarray was analyzed for percentage of positively stained epithelial tissue in each sample by two independent reviewers, including a senior pathologist (AAM), and discrepant values were reconciled by consensus. Statistical testing of mean percentage of positively stained epithelial tissue was performed with two-sided, unpaired student’s t-test.

### 12. RNA extraction for RNA sequencing (RNA-seq)

Cells from the BICR 22 cell line were seeded into six-well plates at 100,000 cells/well and transfected with either CPSF1 siRNA or control siRNA for 16 h. The transfected cells from each well were trypsinized and placed in 96-well plates at 5,000 cells/well and six-well plates at 50,000 cells/well. SCC090 cells transfected with CPSF1 or an empty vector were seeded in 96-well plates at 5,000 cells/well and six-well plates at 50,000 cells/well. Then, cell proliferation was assessed by proliferation assay as described above (section 2.4). RNA was extracted from the cells in six-well plates using the RNeasy plus mini kit (Qiagen, Hilden, Germany) only if growth inhibition of CPSF1-siRNA cells or rapid growth of CPSF1-overexpressed cells was confirmed at 72 h after transfection. RNA extraction was performed in triplicate. RNA samples were required to achieve an RNA integrity number of at least 9.0, an A260/A280 of greater than 1.8, and an A260/A230 of greater than 1.8.

### 13. RNA-seq analysis

Libraries were generated using Illumina’s TruSeq Stranded RiboZero Gold Library prep kits (San Diego, CA), in which ribosomal RNA is removed, followed by a fragmentation step, and reverse transcription with random hexamer primers. Sequencing was performed using the HiSeq 4000 platform sequencer (Illumina) and the TruSeq Cluster Kit. Approximately 80 million 100 × 100 paired-end reads per sample were obtained at the IGM Genomics Center at the University of California San Diego. Subsequently, the RNA-seq data were normalized on the basis of version 2 protocols developed by TCGA [4]. Alignment to the GRCh37/hg19 genome assembly was performed using MapSplice2 version 2.2.1. Junction data from the sequence alignments were extracted for further analysis. Gene expression values were quantified from RNA-seq data using RSEM version 1.2.9 and upper quartile normalization according to the TCGA RSEM v2 normalization pipeline, as previously described [4, 12]. Using these data, the knockdown dataset and overexpression dataset were created for further analyses.

### 14. Analysis of differentially expressed splice junctions

Using the junction data of the knockdown and overexpression datasets, significant genes that had differential splice structures between the conditions were identified through the following steps:

Consider a gene with *n* splice junctions. Each transcript of this gene contains a particular subset of the junctions, which in turn defines a particular correlation structure for junction use. For example, the presence of one junction in a transcript determines with absolute certainty the presence (or absence) of another junction. It is therefore clear that individual junction counts may not be considered independent variables for purposes of significance testing. Rather, it is individual genes represented by *vectors* of junction counts, which may be tested as individual statistical tests. Since genes with multiple exons tend to have multiple transcripts expressed at different levels, the observed junction counts will have a complex covariance structure. This structure can be predicted using the current transcript annotation, but it is of limited use because the transcript expression levels are not known exactly, the well-known positional bias of RNA-seq technology, and other factors such as amplification bias and random sampling.

We proceeded formally as follows: Let a gene’s splice structure be described by a vector of experimentally observed counts

$$\vec{c}=(c_{1},c_{2},\ldots ,c_{n}),$$

where each value is the number of reads spanning the junction. In an experiment with two conditions and *n_r_* replicates, there will be 2*n_r_* count vectors. As we are interested in changes in junction use patterns between conditions, not in changes of gene expression levels, the influence of gene expression levels on junction counts needs to be eliminated. We do this by defining normalized junction patterns as

$$\vec{x}=\frac{\vec{c}}{\sum_{i}c_{i}}.$$

This variable satisfies the constraint ∑*_i_x_i_* = 1, which means the coordinates *x_i_* are *compositional* in nature [15]. This constraint generates an additional source of covariance. For example, in a gene with two junctions, coordinates *x*_1_ and *x*_2_ are perfectly anti-correlated. Following Aitchison [16], we express the composition $\vec{x}$ using the additive log-ratio transformation as

$$\vec{y}=\left(\ln\frac{x_{1}}{x_{n}},\ln\frac{x_{2}}{x_{n}},\cdots ,\ln\frac{x_{n-1}}{x_{n}}\right).$$

Note that $\vec{y}$ has *n*−1 components, which is the number of degrees of freedom in a composition with *n* elements. The choice of the *n*-th element for the denominator was arbitrary but it is irrelevant to the outcome of the statistical testing. We assess the significance of differential splice pattern use between the two experimental conditions as the significance of difference between vectors $\vec{y}$ in the two conditions. To this end, we use Hotelling’s *T*^2^ test [17]. The *T*^2^ test respects the covariate structure of the vector components and estimates the covariance matrix from the data. Hotelling showed that the null distribution of the *T*^2^ statistic (apart from a multiplicative constant) is the $F_{n-1,2n_{r}-n+1}$ distribution, which means that meaningful *p*-values can only be obtained when 2*n_r_−n*+1>0. When the experiment is done with *n_r_* = 3 replicates, one can only assign a *p*-value to genes with fewer than 7 junctions. When a threshold is set for a junction to be considered “in use”, we are only restricted to genes with fewer than 7 junctions in use. In practical terms, when a minimum per condition mean count of 3 is set as the threshold, given the depth of coverage that we have, there are ~70 genes with at least 7 junctions in use. Rather than discarding these genes completely as untestable, we amalgamate small junctions counts into one bin to create a set of 6 junctions (one of which is the amalgamated bin), thus creating a testable hypothesis.

### 15. Validation in biologic samples using qRT-PCR

To validate junction expression from the RNA-seq data, qRT-PCR was performed on cDNA generated from the same cell line RNA used for RNA-Seq. Junction expressions of RNA-seq were normalized by the total junction reads in each sample. Primer sets were designed specifically to span the unique junction using the several significant junctions from the junction analysis. The primers were designed using Primer-BLAST (https://www.ncbi.nlm.nih.gov/tools/primer-blast/) (S1 Table). Touchdown PCR was used to accurately confirm the appropriate length of the PCR product for the primers used.

### 16. Comparison with TCGA cohorts

To validate selected junctions identified in the knockdown datasets, comparisons were performed using junction expression data from TCGA, which were obtained previously. Junctions from the knockdown dataset were compared with the TCGA HPV-negative cohort, because the knockdown dataset was obtained from an HPV-negative cell line (BICR22). Junction expressions were normalized as RPM (reads per million), divided by total gene expression using RSEM and log transformed. Junction expression levels in TCGA cohort were analyzed between normal and tumor and compared with normalized junction expression in the knockdown datasets.

### 17. Presence of the binding site of CPSF1 around ASE associated with CPSF1 overexpression

To confirm the presence of the binding site of CPSF1 (AAUAAA), the top 20 significant ASE was investigated. The binding sequence within 500 bp of the ASE was searched using the DNA sequence of each gene obtained by Ensemble website (ensembl.org/index.html).

### 18. Single sample gene set enrichment analysis (ssGSEA)

Single sample gene set enrichment analysis (ssGSEA) using the RSEM data from the knockdown and overexpression datasets was performed to identify the differentially expressed gene sets between conditions [18]. The GSVA enrichment scores in each sample were calculated by the “GSVA” package of R using the method “ssgesa” [19]. The top 50 gene sets significantly enriched in each dataset were extracted, clustered and visualized using the function “heatmap.2 of the “gplots” package of R.

### 19. Statistical analysis

All experiments were performed in triplicate. The statistical comparisons of the two groups were performed using Student’s t-test. Statistical significance was defined as P < 0.05. All statistical analyses were performed using R version 3.3.3.

## Results

### 1. Spliceosome genes are associated with ASEs

**Fig 1:**
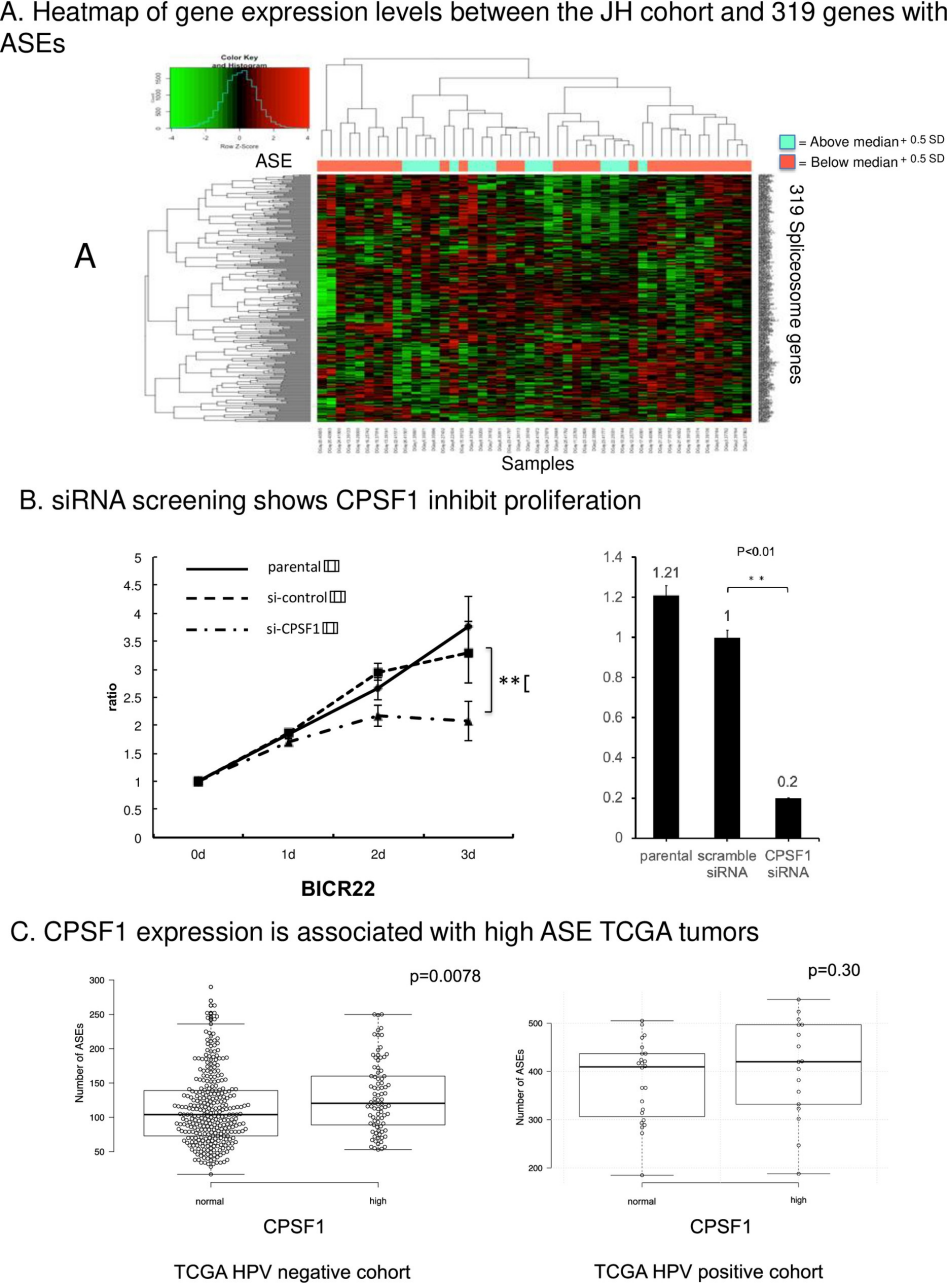
A. Heatmap of gene expression levels between the JH cohort and 319 genes with ASEs. Heatmap and hierarchical clustering of gene expression levels between samples in the Johns Hopkins cohort and 319 genes with alternative splice event numbers in each sample. There was robust correlation between cluster and ASE. As ASE increased, a defined cluster was obtained. 1. B. Representative results of proliferation assay screening. Representative results of proliferation screening assay using the siRNA of 13 candidate genes. Growth curves of BICR22 cells transfected with si-CPSF1 were compared with si-control cells on day 3. P value was calculated using Student’s t-test. *: P < 0.05, **: P < 0.01. These experiments were performed in triplicate in each siRNA. C. Comparison of ASEs between the CPSF1-high and -Normal groups in TCGA. The CPSF1-high group was defined as samples with a Z score > 2 by cBioPortal (http://cbioportal.org). The other samples were defined as the Normal group. Center lines show the medians; box limits indicate the 25th and 75th percentiles as determined by R software; whiskers extend 1.5 times the interquartile range from the 25th and 75th percentiles, outliers are represented by dots. The number of ASEs was significantly different between the Normal- and high-expression CPSF1 groups in TCGA HPV-negative cohort. P value was calculated by using Student’s t-test. **: P < 0.01.

The 319 genes were selected using the QuickGO website (S2 and S3 Tables). To investigate the relationship between the 319 genes and ASEs, a heatmap using unsupervised hierarchical clustering of the gene expression levels with ASE number was created for the samples in an HPV positive HNSCC primary tumor cohort (Fig 1A). Tumors with high ASE was divided two groups median + 0.5SD (Fig 1A). These figures showed that a defined cluster was obtained as ASE increased. We were able to identify a cluster of tumors with high ASE that was associated with spliceosome gene expression. Of note, variation of cutoff thresholds yielded similar results (median, and median + 1.0SD (S1 Fig), We used these data to support a hypothesis that aberrant expression of spliceosome genes is associated with ASE expression in HNSCC, and infer that expression of these genes may also drive a malignant phenotype.

### 2. Determination of the 13 candidate spliceosome genes altered in HNSCC

TCGA data from 279 head and neck cancer samples were used to obtain an overview of the alterations (mutation, CNV, and expression) of the 319 spliceosome genes in HNSCC using cBioPortal (S4 Table). We selected 13 genes (PRPF6, DBR1, PSIP1, HNRNPL, SNRPN, SRPK2, DHX9, YTHDC1, TRA2B, RSRC1, CPSF7, CPSF1, and RBM4) for further analysis based on high number of mutations and/or CNV, with accompanying expression alterations (Table 1). Of note, essentially all of the genes showed alterations consistent with potential oncogenic function, with evidence of amplification and high expression levels, with no candidates identified as potential tumor suppressor gene candidates based on decreased expression in tumor samples.

**Table 1:** Thirteen candidate genes with high number of mutation, copy number variation and expression in TCGA.

Gene symbol	Description	Mutation	Copy Number Variation		Expression		siRNA screening	
		# tumors with	# tumors with	# tumors with	# tumors with	# tumors with		
		Mutation	Amplification	Deletion	High expression	Low expression	Cell line 1	Cell line 2
PRPF6	pre-mRNA processing factor 6	8	1	0	38	8	BICR22	SCC9
DBR1	Debranching RNA Lariats 1	7	29	0	65	0	SCC2	SCC090 (+)
HNRNPL	Heterogeneous Nuclear Ribonucleoprotein L	7	4	0	16	0	SCC11B	SCC17B
PSIP1	PC4 and SFRS1 interacting protein 1	7	11	2	21	0	BICR22	SCC9
SNRPN	Small Nuclear Ribonucleoprotein Polypeptide N	6	2	1	17	0	SCC17B	SCC090 (+)
DHX9	DExH-Box Helicase 9	5	4	0	22	3	Detroit562	BHY
SRPK2	SFRS protein kinase 2	5	13	0	22	1	SCC17B	BICR22
YTHDC1	YTH domain containing 1	5	2	0	13	0	SCC11B	Detroit562
CPSF7	Cleavage And Polyadenylation Specific Factor 7	4	4	0	24	5	SCC090(+)	SCC2
RSRC1	Arginine And Serine Rich Coiled-Coil 1	4	40	0	89	0	SCC090 (+)	93VU147T(+)
TRA2B	Transformer 2 Beta Homolog	4	55	0	50	0	BICR22	BHY
CPSF1	Cleavage And Polyadenylation Specific Factor 1	3	30	0	76	2	BICR22	SCC9
RBM4	RNA Binding Motif Protein 4	3	22	0	34	0	Detroit562	BICR22

These genes were determined based on high levels of mutation, copy number variation and expression in 279 head and neck cancer samples in TCGA. This table includes a summary of the screening. Cell lines were selected from the top 5 expressing cell lines with high expression due to availability in culture. These assays were performed four times: if growth inhibition was observed in at least 3 of the 4 times, it was defined as Growth Inhibition (with a gray background); otherwise, it was defined as not significant (no background). This summary indicates that CPSF1 and YTHDC1 effectively inhibited cell growth.

### 3. Screening using proliferation assays following siRNA knockdown of 13 genes

Proliferation assays were selected for screening to determine potential driver genes among the 13 candidate genes, using appropriate HNSCC cell lines with alteration in gene specific expression. The mRNA expression data of the OPC-22 panel used by Martin et al. [13] was referred to in selecting cell lines (S5 Table). Cell lines with the top five highest mRNA expression levels of a candidate gene were selected for each gene, and two cell lines for each gene were selected and used for this assay. All siRNA knockdown were validated by RT-PCR. Specific siRNA for each gene were selected, and growth curves were compared between the si-control and si-target gene knockdown cells. (Table 1). The siCPSF1 effectively inhibited cell growth, while other siRNAs for other gene candidates did not show significant or consistent growth inhibition (Fig 1B). The CPSF1 siRNA knockdown was also validated by qPCR (Fig 1B). Additionally, growth inhibition by CPSF1 knockdown was validated using other separate siRNA (S2 Fig). In the experiments using the BICR22 cell line, siCPSF1 significantly inhibited cell proliferation compared with the si-control.

### 4. Overexpression of CPSF1 is associated with increased levels of ASEs in HPV-negative HNSCC

To determine whether CPSF1 are associated with a high ASE phenotype in primary tumors, the total number of ASEs in each sample was counted using the same method previously described for the TCGA HPV-positive and HPV-negative cohorts [12]. First, we performed an outlier analysis to identify the significant variance using the HPV-positive and -negative TCGA tumors (407 HPV-negative tumors, 90 HPV-positive tumors and 44 normal samples). We found 580 ASEs in the TCGA HPV-negative cohort and 210 ASEs in the TCGA HPV-positive cohort (S7 Table) [14]. Then, the numbers of ASEs in each sample were measured (S8 Table). Afterward, a box plot was created to examine the relationship between ASE number and CPSF1 expression level in TCGA tumors (Fig 1C). This figure indicates that the CPSF1-overexpression subset had a significantly high number of ASEs in the HPV-negative TCGA cohort.

### 5. Knockdown of CPSF1 by inducible shRNA induces apoptosis and inhibits cell proliferation and colony formation

**Fig 2:**
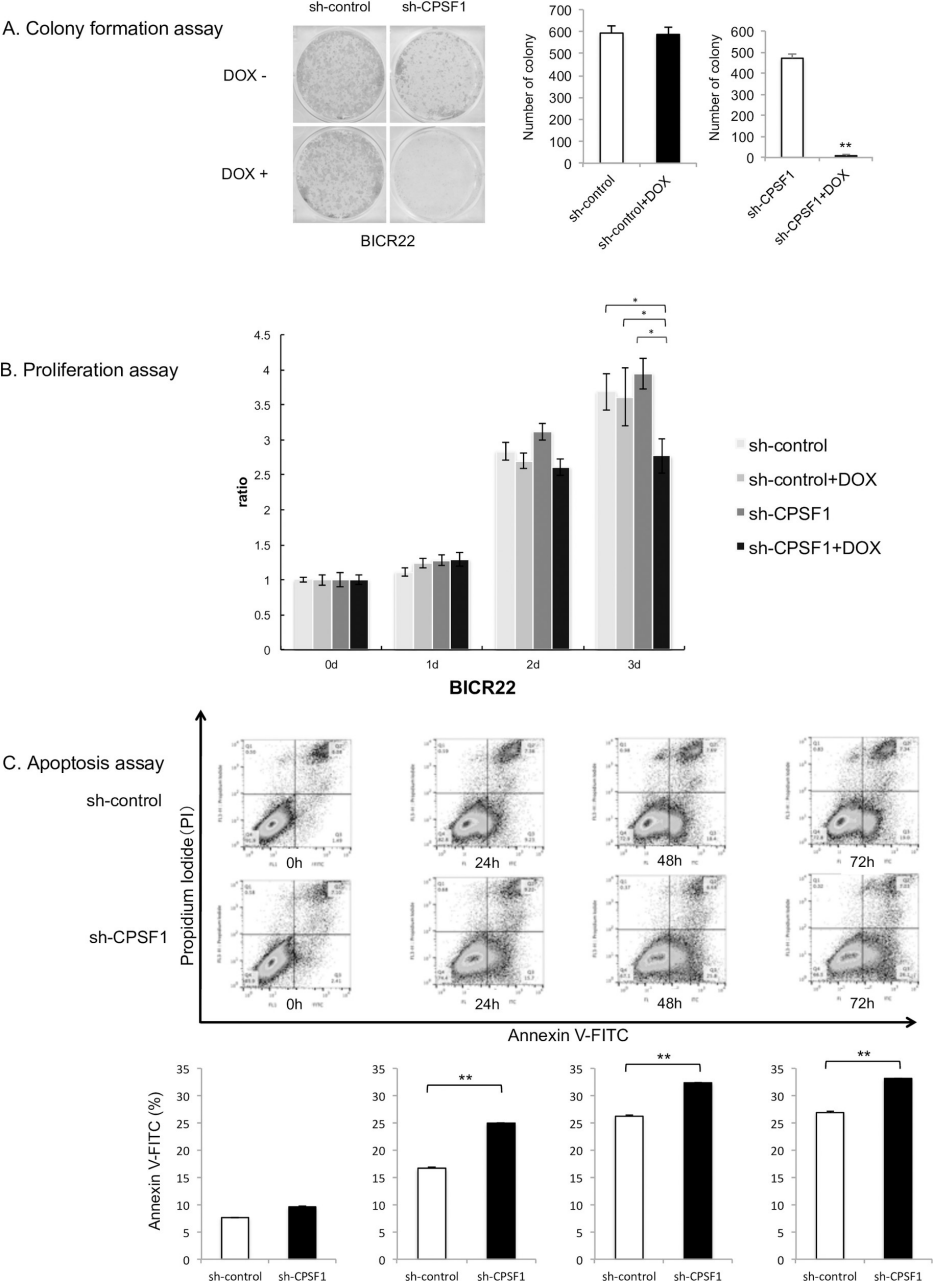
A. Colony formation assay. Knockdown of CPSF1 by inducible sh-CPSF1 decreased the colony formation ability of BICR22 cells. Cells containing sh-control and sh-CPSF1 were incubated for 14 days with or without DOX. The number of colonies formed in the sh-CPSF1 cells with DOX was significantly decreased compared with the sh-control cells. These experiments were performed in triplicate. P value was calculated using Student’s t-test. *: P < 0.05, **: P < 0.001. B. Results of the proliferation assay. Cell proliferation was detected in the BICR22 cell lines after stable transfection of inducible sh-control or sh-CPSF1. The proliferation of the cells with sh-CPSF1 and DOX was inhibited compared to other cells. A two way ANOVA was conducted to compare the main effects and interaction between use of DOX and cell line. There as a significant main effect of use of DOX (p<0.001). Also, there was a significant interaction between DOX and cell lines (p<0.01). As the interaction was significant, Post hoc analyses was performed as a multiple comparison of all groups. Post hoc analyses using Steel-Dwass test showed that CPSF1+ DOX group had a significant difference compared to other three groups (p<0.05). These experiments were performed in triplicate. *: P < 0.05. C. Apoptosis assay. Apoptotic cells were measured three times after induction with 1 μg/ml DOX in sh-control- and sh-CPSF1-cells by flow cytometric analysis. Apoptosis in the sh-CPSF1 cells was significantly increased compared with the sh-control cells. P value was calculated using Student’s t-test. *: P < 0.05, **: P < 0.001.

To further investigate the function of CPSF1, we created a stable, inducible CPSF1 shRNA cell line (sh-CPSF1). The knockdown of CPSF1 was validated by qRT-PCR and Western blot (S3 Fig). Colony formation assays (Fig 2A) show a dramatic decrease in number of colonies in sh-CPSF1 cells in the presence of doxycycline in comparison to control cells. Similarly, the proliferation of sh-CPSF1 cells in the presence of doxycycline was limited compared with that of the other cells (Fig 2B), and apoptosis in the sh-CPSF1 cells was significantly increased compared with that in the sh-control cells (Fig 2C).

### 6. Overexpression of CPSF1 induces cell proliferation and tumorigenicity

**Fig 3:**
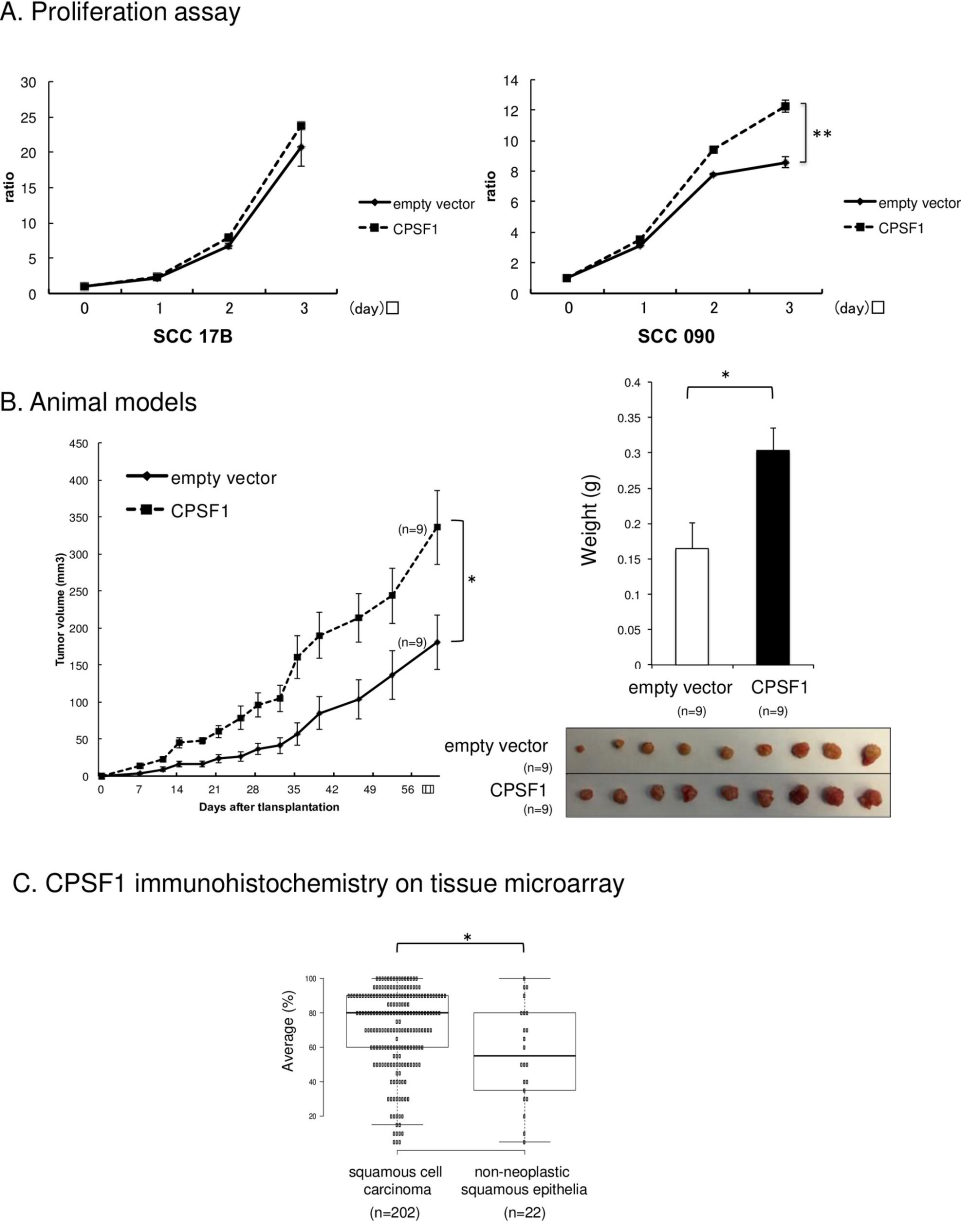
A. Overexpression of CPSF1 promoted cell proliferation. Cell proliferation was detected in SCC17B and SCC090 cell lines after transfection of empty vector and CPSF1 vector. The proliferation of the SCC090 cells overexpressing CPSF1 was significantly increased compared to empty vector cells. P value was calculated using Student’s t-test. *: P < 0.05, **: P < 0.001. These experiments were performed in triplicate. B. Overexpression of CPSF1 induced tumorigenicity. SCC090 cells with the empty vector and overexpression of CPSF1 were injected into nude mice and examined for tumorigenic ability. Tumor progression in the nude mice injected with cells overexpressing CPSF1 was faster than that in the nude mice inoculated with empty-vector-transfected cells. Tumor weight in the CPSF1 group was significantly heavier than in the empty vector group. P value was calculated using Student’s t-test. *: P < 0.05. These experiments were performed in triplicate. C. CPSF1 immunohistochemistry on tissue microarray. C shows the average of CPSF1 expression by IHC staining between non-neoplastic epithelia and squamous cell carcinoma cores from the tissue microarray. Immunohistochemistry of the tissue microarrays demonstrated statistically significant overexpression of CPSF1 in head and neck squamous cell carcinoma tumor as compared to non-neoplastic squamous epithelial tissue (p = 0.016). P value was calculated using Student’s t-test. *: P < 0.05.

To examine the oncogenic potential of CPSF1, CPSF1 was stably transfected into the two cell lines (SCC17B and SCC090) that had the lowest mRNA expression levels of CPSF1 among the cell lines used for screening, to create CPSF1 overexpressing cell lines. The overexpression of CPSF1 was validated by qRT-PCR and Western blot (S4 Fig). We examined growth curves to investigate the effect of overexpression on proliferation (Fig 3A) The proliferation of SCC090 cells overexpressing CPSF1 significantly increased compared with that of the empty vector cells but the proliferation increase in SCC17B cells was not significant. To elucidate the carcinogenic effect of CPSF1, we employed a nude mouse xenograft model and injected SCC090 cells overexpressing CPSF1 into nude mice and examined their tumorigenic ability. Tumor growth in the nude mice injected with cells overexpressing CPSF1 was increased compared to empty vector-containing cells (Fig 3B). We also found that the tumor volumes in the CPSF1 group were significantly larger than those in the empty vector group (p<0.05) at all time points (Fig 3B). The overexpression of CPSF1 in tumor was confirmed by qRT-PCR and Western blot (S5 Fig).

### 7. CPSF1 is overexpressed in primary HNSCC

Immunohistochemistry of the tissue microarray, which included 22 cores of non-neoplastic squamous epithelium and 202 cores of head and neck squamous cell carcinoma representing a broad distribution of primary sites, demonstrated statistically significant overexpression of CPSF1 in head and neck squamous cell carcinoma tumor as compared to non-neoplastic squamous epithelial tissue (p = 0.016) (Fig 3C). S6 Fig shows representative non-neoplastic epithelia and squamous cell carcinoma cores from the tissue microarray with CPSF1 IHC staining.

### 8. CPSF1 promotes aberrant splicing of cancer-associated genes

To identify the genes that have significant differential splice structures after knockdown or overexpression of CPSF1, junction analysis was performed using the knockdown and overexpression datasets. A knockdown dataset was created using the junction data defined using Mapsplice2 and gene expression data by RSEM from RNA-seq data of the CPSF1 knockdown cell line BICR22 (HPV negative cell line). Thirty-nine genes were identified as having significantly differential splicing (Table 2 and S9 Table). These included many cancer-associated genes such as AKT2, HRAS, TGFBI and UBE2C. The overexpression dataset was created using the data from the SCC090 CPSF1 stably overexpressing cell line. Fifty-six genes were identified as significant genes and included cancer-associated genes such as BOK, FANCD2, ADRM1 and EGLN1 (Table 3 and S10 Table). Moreover, the binding sequence of CPSF1 were identified around the junctions in these significant genes (S11 Table). We also investigated the overlapping genes that significantly changed in both knockdown and overexpression of CPSF1 and found that two genes, UBE2C and SZT2 were overlapped (S7 Fig). We were able to define a specific SZT2 splice junction that were decreased in the CPSF1 knockdown dataset with a reciprocal increase in expression in the CPSF1 overexpression dataset as well as SZT2 splice junctions that were increased in the CPSF1 knockdown dataset and decreased in the CPSF1 overexpression dataset.

**Table 2:** The list of top 20 significant genes after junction analysis of the CPSF1 knockdown dataset.

Gene symbol	Gene Name	p value
PNISR	PNN interacting serine and arginine rich protein(PNISR)	0.000035
SLC39A1	solute carrier family 39 member 1(SLC39A1)	0.00041
LAMC2	laminin subunit gamma 2(LAMC2)	0.00061
UBE2C	ubiquitin conjugating enzyme E2 C(UBE2C)	0.0037
PUM2	pumilio RNA binding family member 2(PUM2)	0.0057
SLC5A6	solute carrier family 5 member 6(SLC5A6)	0.0091
HRAS	HRas proto-oncogene, GTPase(HRAS)	0.01
AKT2	AKT serine/threonine kinase 2(AKT2)	0.011
HDLBP	high density lipoprotein binding protein(HDLBP)	0.011
TK1	thymidine kinase 1(TK1)	0.011
TXN2	thioredoxin 2(TXN2)	0.011
MIR4435-1HG	MIR4435-2 host gene(MIR4435-2HG)	0.013
EIF4G1	eukaryotic translation initiation factor 4 gamma 1(EIF4G1)	0.015
NIN	ninein(NIN)	0.015
MICAL2	microtubule associated monooxygenase, calponin and LIM domain containing 2(MICAL2)	0.017
PIGO	phosphatidylinositol glycan anchor biosynthesis class O(PIGO)	0.019
PRMT5	protein arginine methyltransferase 5(PRMT5)	0.019
ZNF618	zinc finger protein 618(ZNF618)	0.024
MIR205HG	MIR205 host gene(MIR205HG)	0.027
UBR3	ubiquitin protein ligase E3 component n-recognin 3 (putative)(UBR3)	0.028

**Table 3:** The list of top 20 significant genes after junction analysis of the CPSF1 overexpression dataset.

Gene symbol	Gene name	p value
BOK	BOK, BCL2 Family Apoptosis Regulator	0.00065
MAP4	Microtubule Associated Protein 4	0.00065
ATG13	Autophagy Related 13	0.0018
FANCD2	Fanconi anemia, complementation group D2	0.0019
AKNAD1	AKNA Domain Containing 1	0.0023
SZT2	SZT2, KICSTOR Complex Subunit	0.0035
MB21D1	Mab-21 Domain Containing 1	0.0046
BPNT1	3'(2'), 5'-Bisphosphate Nucleotidase 1	0.0059
CTC-432M15.3	ENSG00000273217 Gene	0.0061
SLC25A19	Solute Carrier Family 25 Member 19	0.0065
AP1G1	Adaptor Related Protein Complex 1 Gamma 1 Subunit	0.0068
POLR3E	RNA Polymerase III Subunit B	0.009
ZDHHC23	Zinc Finger DHHC-Type Containing 23	0.0093
RUVBL1	RuvB-like 1 (E. coli)	0.0094
MTUS1	Microtubule Associated Scaffold Protein 1	0.01
TRIB3	Tribbles Pseudokinase 3	0.013
ACSL5	Acyl-CoA Synthetase Long-Chain Family Member 5	0.014
PYGO2	Pygopus Family PHD Finger 2	0.014
ELAVL1	ELAV Like RNA Binding Protein 1	0.015
NEK9	NIMA (never in mitosis gene a)- related kinase 9	0.015

### 9. Validation of junction expression with biological samples and a TCGA cohort

**Fig 4:**
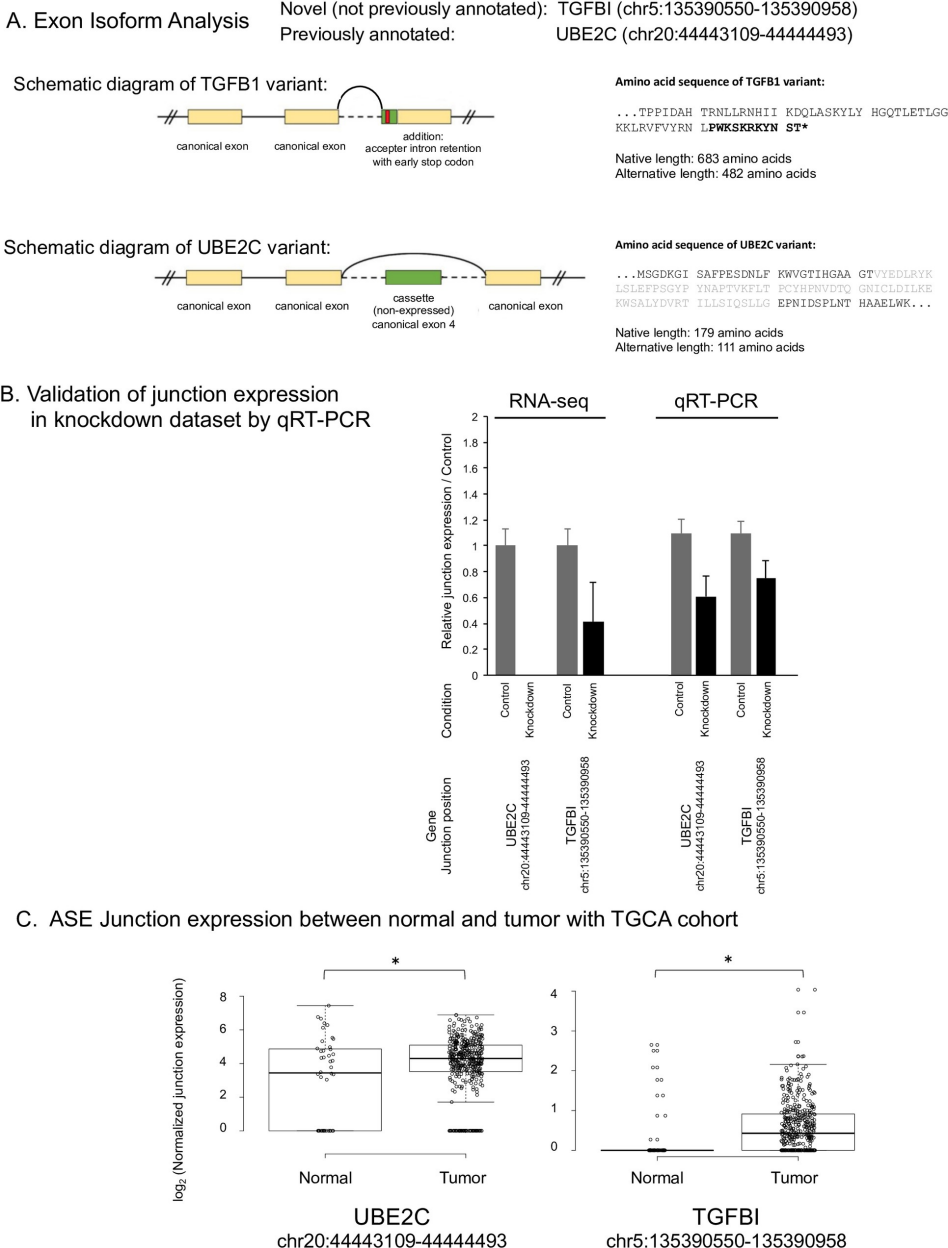
A. Exon Isoform analysis. To validate the junction expression in the knockdown datasets, two candidate junctions (TGFBI (chr5:135390550–135390958) and UBE2C (chr20:44443109–44444493)) in significantly differentially spliced genes were analyzed. TGFB1 variant was novel and UBE2c variant was previously annotated. Each of these alternate splice events is predicted to alter the peptide sequence within the expressed exons: chr5:135390550–135390958 represents accepter intron retention from an early splice accepter site that introduces an early stop codon in TGFB1; chr20:44443109–44444493 introduce cassette exons, which each skips a single canonically expressed exon in UBE2C. B. Validation of junction expression in knockdown dataset by qRT-PCR. To validate the junction expression between conditions in the knockdown datasets, UBE2C (chr20:44443109–44444493) and TGFBI (chr5:135390550–135390958) were selected for RT-PCR. The junction expression of UBE2C and TGFBI in RNA-seq were decreased after knockdown of CPSF1. The change obtained from RNA-Seq was also confirmed by qRT-PCR using same cell line.C. Junction expression between normal and tumor sample with TCGA HPV negative cohorts. Validation using the junction expression data from TCGA HPV negative cohort was performed. A box plot was created to examine the relationship between normal samples and tumor samples in TCGA. In the comparison of junction expression between normal and tumor in TCGA HPV negative cohort, a significant increase of junction expression in tumor was confirmed if this junction expression was decreased in the knockdown cell line.

To validate the junction expression in the knockdown datasets, candidate significantly differentially spliced genes were analyzed. We validated junctions from the cancer-associated genes UBE2C (chr20:44443109–44444493), and TGFBI (chr5:135390550–135390958) which were confirmed by the Integrative Genomics Viewer (IGV) using raw RNA-Seq data (S8 Fig). For each of these alternate splice events, the dominant isoform of its gene was used to specify canonical exon and intron start and stop sites. The exon changes effected by the introduction of each alternate splice site were then manually extrapolated by comparing the alternate with canonical exon start and stop sites. As illustrated in the schematic diagrams (Fig 4A), each of these alternate splice events is predicted to alter the peptide sequence within the expressed exons: chr5:135390550–135390958 represents accepter intron retention from an early splice accepter site that introduces an early stop codon in TGFB1; chr12:112304035–112306557 introduce cassette exons, which skip a single canonically expressed exon in UBE2C.

Junction expression in the knockdown dataset is shown in Fig 4B. The junction expression of UBE2C and TGFBI in RNA-seq were decreased after knockdown of CPSF1. The change obtained from RNA-Seq was confirmed by qRT-PCR using same cell line RNA. Moreover, to validate the junction expression, several other junctions (MICAL2 (chr11:12248678–12260983), WNK1 (chr12:1006847–1017013), ZMYM2 (chr13:20635364–20638591), ADRM1 (chr20:60879541–60881253) and MAPKAPK5 (chr12:112304035–112306557)) were also selected for RT-PCR. The changes of these junction expressions were also confirmed by RT-PCR (S9 Fig). Afterward, validation using the junction expression data from TCGA HPV negative cohort was performed. A box plot was created to examine the relationship between normal samples and tumor samples in TCGA in each junction. The junction expressions of UBE2C and TGFBI in the TCGA HPV negative cohort were shown in Fig 4C. These junctions were selected by the knockdown dataset, which was obtained from the data of the HPV negative cell line (BICR22). Therefore, the validation of these junctions was performed using HPV negative cohort. In the comparison of junction expression between normal and tumor in TCGA HPV negative cohort, a significant decrease in junction expression in normal tissue was confirmed. The gene expression of CPSF1 in the tumor samples in the TCGA HPV negative samples and positive samples was higher than normal samples (S10 Fig). In the CPSF1 knockdown dataset, the junction expressions of UBE2C and TGFBI were decreased by knockdown of CPSF1 as expected.

### 10. CPSF1 expression dysregulation alters cancer-associated gene sets

Given the likelihood that dysregulation of a spliceosome component will have effects on a large number of gene targets via broad alteration of ASEs, specific analysis of the aggregate functional effects of these alterations would be challenging. To identify the gene sets that correlated with CPSF1 dysregulation, we performed genome wide single sample gene set enrichment analysis (ssGSEA) based on total gene expression defined by RSEM using the knockdown and overexpression datasets. The top 50 significant gene sets between conditions in each dataset are shown in S9 and S10 Tables. Six significant cancer-related gene sets were found in the CSFI knockdown dataset including two well-known pathways, metastasis and RAS activation (S11A Fig). Twelve cancer-associated gene sets were identified in the CPSF1 overexpression dataset, including MISHRA_CARCINOMA_ASSOCIATED_FIBROBLAST_UP, RICKMAN HEAD_AND_NECK_CANCER_D, and SMID_BREAST_CANCER_RELAPSE_IN_LUNG_UP (S11B Fig). A well-known TP53 pathway, GALI_TP53_TARGETS_APOPTOTIC_DN, was found in this dataset as well. These data confirm that the net effect of CPSF1 dysregulation results in downstream alterations in target gene sets associated with carcinogenesis.

## Discussion

A number of classic ASEs that are associated with cancer development have been described. For example, bcl-x contains a bcl-xs splice variant with proapoptotic effects and a bcl-xl splice variant with anti-apoptotic effects [6]. There are also examples of spliceosome genes that have been shown to drive carcinogenesis. For example, splicing is regulated by ASF/SF2 (SRSF1), which directly binds and regulates target mRNAs and globally regulates apoptosis [20]. Ron (MST1R) is a tyrosine kinase receptor that shares with the members of its subfamily (Met and Sea) and can control cell dissociation, motility, and invasion [7]. Ron has an isoform called dRON, which is generated by alternative splicing through the skipping of exon 11 and is expressed in breast and colon cancer [21]. dRON is also regulated by SRSF1 and plays a role in regulating malignant transformation by inducing epithelial-mesenchymal transition (EMT) [22].

We hypothesized that the genomic alteration of genes associated with the spliceosome may broadly induce ASEs across a broad range of target genes, driving an oncogenic phenotype. We identified 319 spliceosome genes and employed a discovery pipeline to identify 13 candidate spliceosome genes altered in HNSCC using TCGA HNSCC data. Proliferation assay using siRNAs of 13 candidate genes showed that CPSF1 effectively inhibited cell growth and that they could be modulator genes. To examine the relevance to ASEs, we investigated the relationship between ASE and CPSF1 expression levels and revealed that the overexpression of CPSF1 increased the number of ASEs. Of note, one would expect that there are multiple potential factors that may influence splicing alteration, so the influence of other genes on ASE frequency would be expected. This does not mean that CPSF1s biologic effects are negated, but they are likely modulated by more complex interactions with other regulatory pathways that affect ASE. Therefore, we conclude that CPSF1 has the potential to affect ASE expression and drive proliferation in HNSCC.

Cleavage and polyadenylation specificity factor (CPSF) is the central component of the 3’ processing machinery, and CPSF1 is the largest subunit of the CPSF complex [23]. Burge et al. showed that proliferating cells exhibited genome-wide truncation of mRNA structure (especially 3'UTRs) [5]. Kiehl et al. [24] reported that a significant increase in CPSF1 expression was detected in lung cancer samples in comparison with normal lung, and it suppressed the RASSF1A tumor suppressor gene, which is epigenetically inactivated in a wide range of cancer types [25]. Van Etten et al. [26] reported that CPSF1 regulates Androgen receptor (AR) splice variant expression in prostate cancer.

We found the overexpression of CPSF1 increased the tumorigenicity of the head and neck cancer cells using adherent cell line models as well as *in vivo* using xenograft models. Furthermore, to confirm the overexpression of CPSF1 in head and neck cancer primary tumors, we performed immunohistochemistry on as separate tissue microarray. These results demonstrated statistically significant overexpression of CPSF1 in HNSCC as compared to non-neoplastic squamous epithelial tissue. In summary, we demonstrated that CPSF1 overexpression is a candidate oncogenic event in head and neck cancer.

We hypothesized that the aberrant expression of CPSF1 changed the ASEs, thereby conveying the ability to induce oncogenesis. Previously, microarrays [27] [11] or high-throughput RT-PCR [28] was used to identify ASEs that differed between normal and cancer samples. However, along with the recent development in next-generation sequencing technology, RNA-Seq is widely used to analyze the transcriptome [29, 30] [31]. We conducted RNA-Seq from the knockdown cell lines and overexpression cell lines of CPSF1 and junction analysis was performed to detect the genes with ASEs in each dataset. In particular, genes known to be associated with cancer such as LAMC2, UBE2C, AKT2, AKT2, BOK, MAP4, and FANCD2 were noted to be aberrantly spliced, indicating that the aberrant expression of CPSF1 significantly altered the ASEs of oncogenes in each dataset. Then, to validate the junction expression levels from the RNA-Seq data, qRT-PCR was performed. We selected 2 genes (UBE2C, TGFBI) with junctions that were associated with cancer and had overlap with junction data from the TCGA cohort for validation. Overexpression of UBE2C has been detected in many types of human cancers[32], and TGFBI is a candidate regulator of EMT[33]. However, the functions of these splice variants has not been clarified. Exon analysis showed that the junctions of TGFBI (chr5:135390550–135390958) is novel and likely change protein function. The change in the junction expression obtained from the RNA-Seq between conditions was also confirmed by qRT-PCR using the cell line RNA. In the comparison of junction expression between normal and tumor in each TCGA cohort, a significant increase of junction expression in tumor was confirmed as we expected. These results also indicated the validity of our junction data. Here we demonstrated that aberrant expression of CPSF1 induces ASE, but the correlation between activation of CPSF1 and mutation or epigenetic changes including methylation has not been investigated. Further consideration will be needed to explore additiona findings related mutation or epigenetic alterations driving CPSF1 expression.

Finally, we performed ssGSEA analysis in each dataset to determine the gene sets altered by aberrant CPSF1 expression, and defined multiple cancer-associated gene sets in association with CPSF1 dysregulation. These results didn’t indicate that it was caused solely by splicing. However, CPSF1 regulates splicing. Therefore, there is a possibility that gene expression changes caused by aberrant CPSF1 are associated with splicing.

Given the complexity of ASE expression, and the broad effects on ASE expression across multiple genes, we were not able to define a single splice variant that executes the phenotypic effects that result from CPSF1 overexpression. However, given the central role of CPSF1 in the spliceosome and processing of the 3 prime UTR, it is interesting that the splice variants defined by CPSF1 dysregulation included ASEs outside the UTR. It is perhaps not unexpected that broad alterations in ASE expression were noted in our model systems, directly reflecting the large repertoire of ASEs associated with CPSF1 in primary tumors. It is possible that CPSF1 initiates downstream effects mediated through other genes, either through direct interaction or through spliceosome dysregulation that affects other splicing mechanisms, or secondary and indirect effects on other genes. Further studies may be required to elucidate the relative functional contributions of specific splice variants related to CPSF1 in HNSCC.

## Supporting information

S1 Fig A. Heatmap of gene expression levels between the JH cohort and 319 genes with ASEs divided by median. B. Heatmap of gene expression levels with ASEs divided by median + 1.0 SD. Variation of cutoff thresholds yielded similar results (median, and median + 1.0SD). C. Heatmap of gene expression levels with ASEs using supervised clustering.
(TIF)

S2 Fig Proliferation assay using several siRNAs of CPSF1. Growth inhibition by CPSF1 knockdown was found using other separate siRNAs. P value was calculated using Student’s t-test. *: P < 0.05, **: P < 0.01.
(TIF)

S3 Fig Validation of knockdown by qRT-PCR and Western blot. The mRNA expression level of CPSF1 in BICR22 cells transfected by sh-control and sh-CPSF1. Total RNA was collected 2 days after induction with 1 μg/ml doxycycline (DOX). qRT-PCR shows that the expression level of CPSF1 in sh-CPSF1 cells was significantly lower than that in sh-control cells. Protein was collected 3 days after induction with 1 μg/ml doxycycline (DOX). The Western blot showed that the protein expression of CPSF1 in sh-CPSF1 was decreased compared with the sh-control.
(TIF)

S4 Fig Validation of overexpression by qRT-PCR and Western blot. The mRNA expression level of CPSF1 in the SCC17B and SCC090 cell lines transfected with empty vector or CPSF1 overexpression vector. qRT-PCR showed that the expression of CPSF1 in SCC17B and SCC090 cell lines after transfection with CPSF1 was significantly higher than in empty vector cells. The Western blot showed that the protein expression of CPSF1 was overexpressed compared with the empty vector.
(TIF)

S5 Fig Validation of overexpression in the tumor of the mice by qRT-PCR and Western blot. CPSF1 overexpression was validated by Western blot and qRT-PCR. CPSF1 expression in the tumor of overexpressed CPSF1 group was higher than empty vector group by qRT-PCR and Western blot.
(TIF)

S6 Fig CPSF1 immunohistochemistry on tissue microarray. Representative non-neoplastic epithelia and squamous cell carcinoma cores from the tissue microarray with CPSF1 immunohistochemistry staining. Overexpression of CPSF1 in head and neck squamous cell carcinoma tumor was seen compared to non-neoplastic squamous epithelial tissue.
(TIF)

S7 Fig Junction expressions of SZT2 and UBE2C by Junction analysis. Some junction expressions in SZT2 were decreased in knockdown dataset and increased in overexpression dataset or increased in knockdown dataset and decreased in overexpression dataset. However, junction expressions in UBE2C didn’t show the reversed changes between two datasets.
(TIF)

S8 Fig Junction expressions of UBE2C and TGFBI by IGV. Junction expression of UBE2C (chr20:44443109–44444493) and TGFBI (chr5:135390550–135390958) were confirmed by Sashimi plot on the Integrative Genomics Viewer (IGV) using raw RNA-Seq data. Junctions were decreased by knockdown of CPSF1.
(TIF)

S9 Fig Validation of junction expression by rRT-PCR. To validate the junction expression, several junctions (MICAL2 (chr11:12248678–12260983), WNK1 (chr12:1006847–1017013), ZMYM2 (chr13:20635364–20638591), ADRM1 (chr20:60879541–60881253) and MAPKAPK5 (chr12:112304035–112306557)) were selected for RT-PCR. The changes of these junction expressions were also confirmed by RT-PCR.
(TIF)

S10 Fig The gene expression of CPSF1 in TCGA HPV negative and positive cohort. The gene expression of CPSF1 in TCGA HPV negative and positive cohort.　In the comparison of CPSF1 gene expression between normal and tumor in each TCGA HPV negative and positive cohort, a significant high gene expression in tumor was confirmed. P value was calculated using Student’s t-test. **: P < 0.001.
(TIF)

S11 Fig A Top 50 significant pathways of Knockdown dataset by ssGSEA. Six significant cancer-related gene sets were found in the knockdown dataset, including two well-known pathways, such as metastasis or RAS activation.B Fig. Top 50 significant pathways of Overexpression dataset by ssGSEA. Twelve cancer-associated gene sets were identified in the overexpression data set. A well-known TP53 pathway, GALI_TP53_TARGETS_APOPTOTIC_DN, was found in this dataset as well.
(TIF)

S1 Table Primer sets for the validation of junction expression. (XLSX)

S2 Table The list of 319 spliceosome genes. (XLSX)

S3 Table 319 genes annotated with spliceosome gene ontology terms. (XLSX)

S4 Table Overview of the alterations (mutation, CNV, and expression) of the 319 spliceosome genes. (XLSX)

S5 Table The mRNA expression data of the OPC-22 panel. (XLSX)

S6 Table The mutational status and copy number variation for interest genes in each cell line we used. (XLSX)

S7 Table Significant splicing variants in TCGA HPV-negative and -positive cohorts by outlier analysis. (XLSX)

S8 Table The number of ASEs in each sample of the TCGA cohort. (XLSX)

S9 Table The list of significant genes after junction analysis of the knockdown dataset. (PDF)

S10 Table The list of significant genes after junction analysis of the overexpression dataset. (PDF)

S11 Table Presence of the binding site of CPSF1 around ASE associated with CPSF1 overexpression. (PDF)

S1 Raw images (PDF)

S2 Raw images (PDF)

S3 Raw images (PDF)
