# Supplementary figures and images for: Aberrant expression of CPSF1 promotes head and neck squamous cell carcinoma via regulating alternative splicing
Source: PLoS One. 2020 May 21;15(5):e0233380. doi: 10.1371/journal.pone.0233380 (PMC7241804; doi:10.1371/journal.pone.0233380)

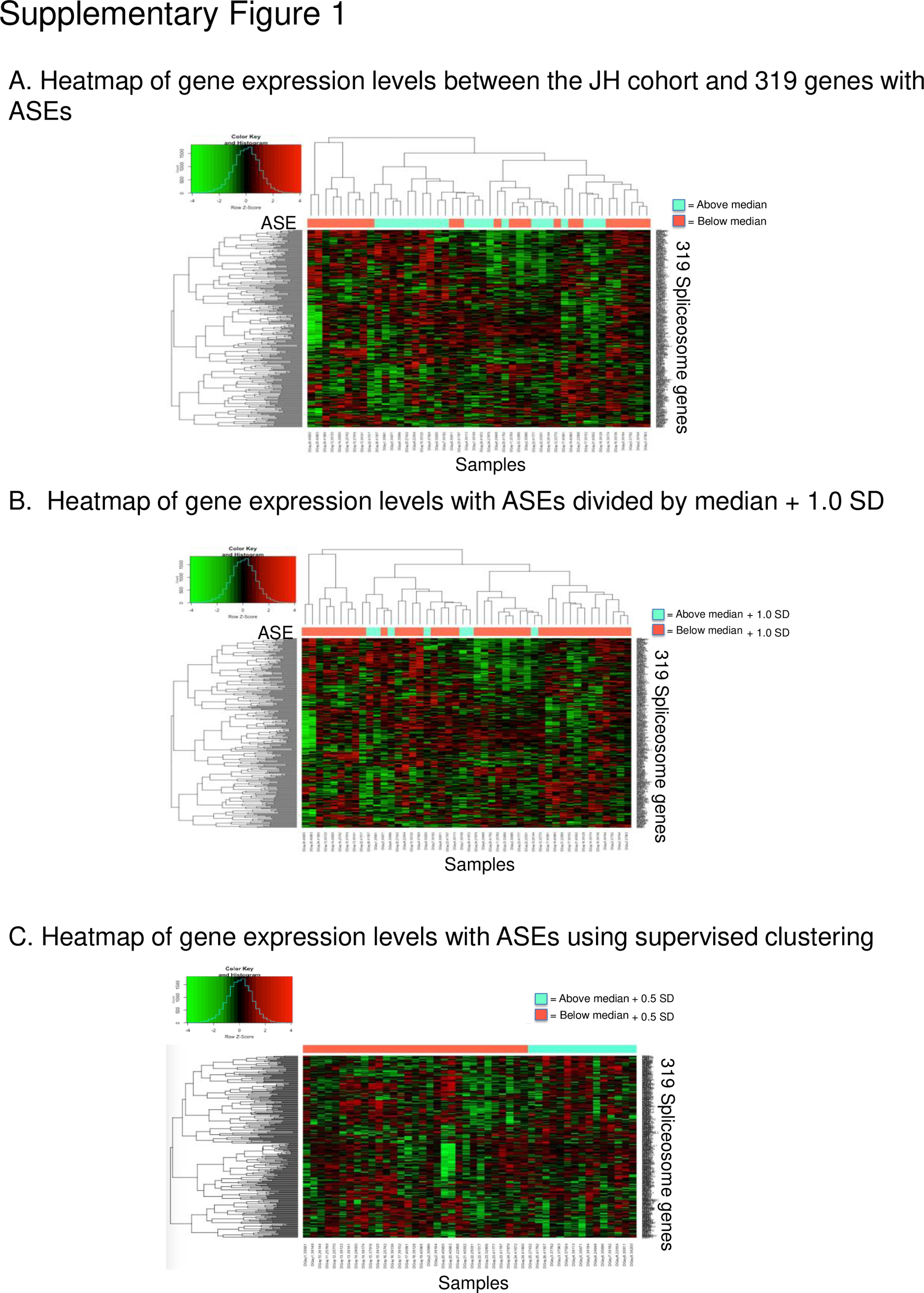

Supplement: S1 Fig — A. Heatmap of gene expression levels between the JH cohort and 319 genes with ASEs divided by median. B. Heatmap of gene expression levels with ASEs divided by median + 1.0 SD. Variation of cutoff thresholds yielded similar results (median, and median + 1.0SD). C. Heatmap of gene expression levels with ASEs using supervised clustering. (TIF) [file pone.0233380.s001.tif]

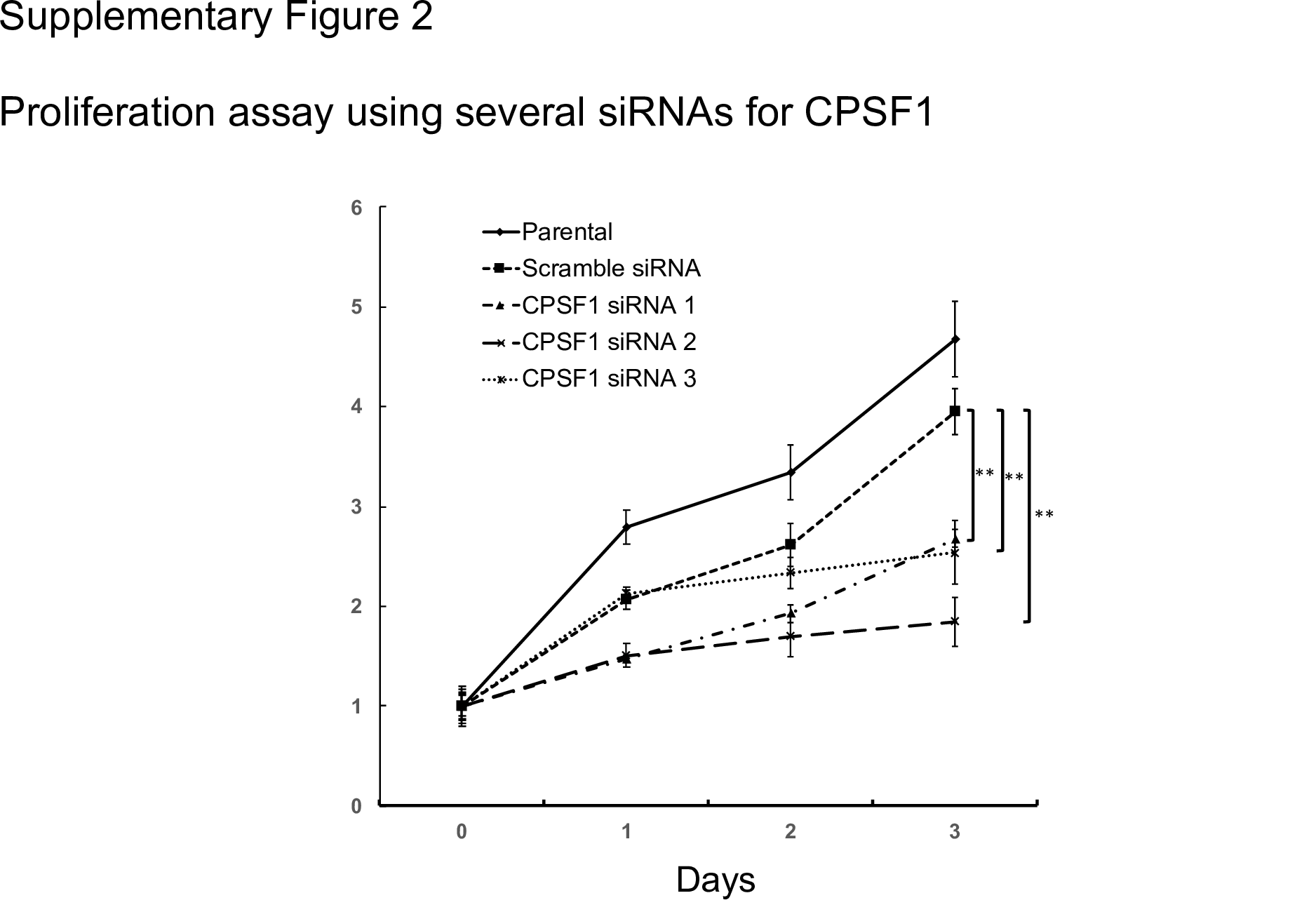

Supplement: S2 Fig — Growth inhibition by CPSF1 knockdown was found using other separate siRNAs. P value was calculated using Student’s t-test. *: P < 0.05, **: P < 0.01. (TIF) [file pone.0233380.s002.tif]

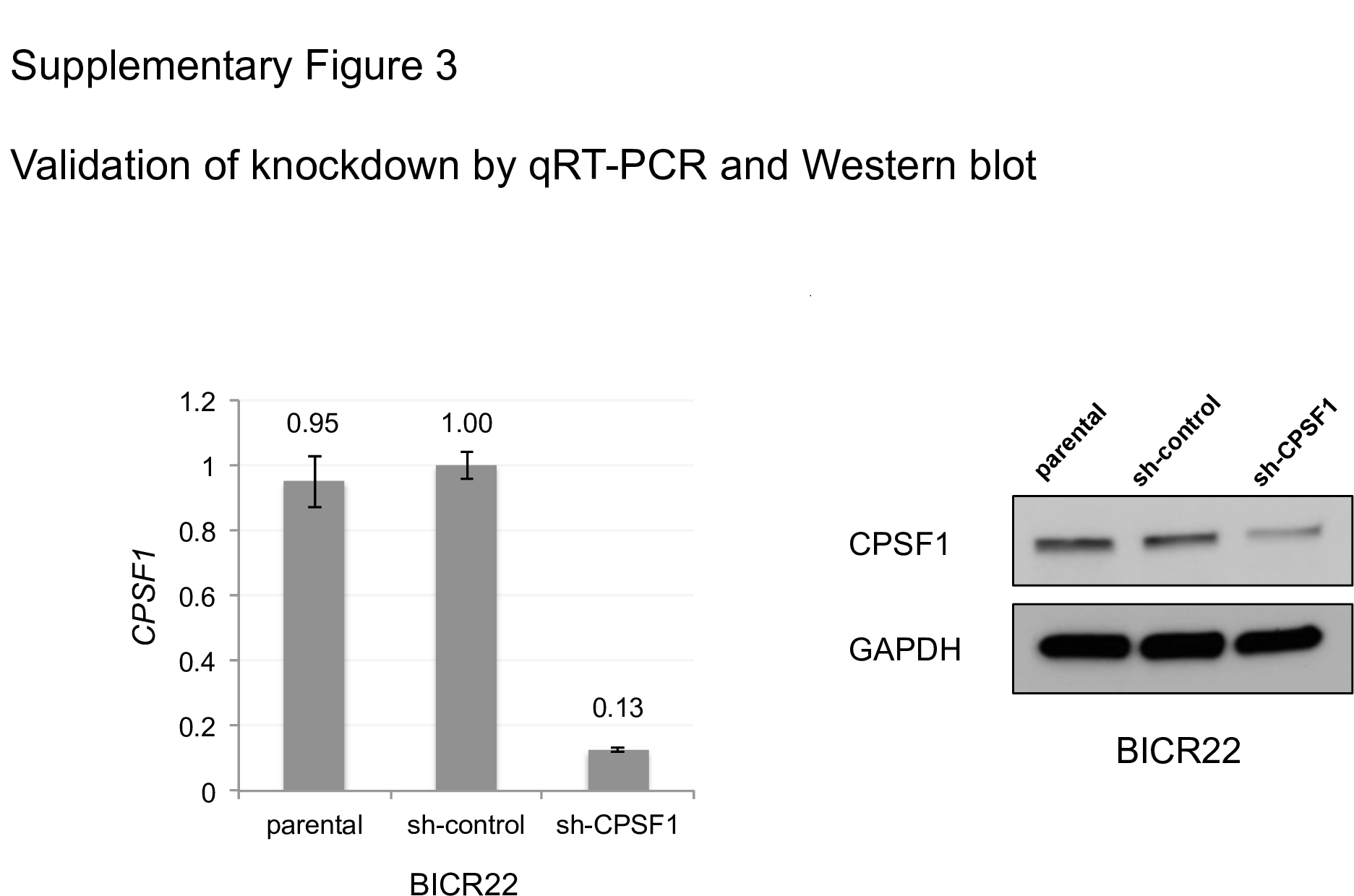

Supplement: S3 Fig — The mRNA expression level of CPSF1 in BICR22 cells transfected by sh-control and sh-CPSF1. Total RNA was collected 2 days after induction with 1 μg/ml doxycycline (DOX). qRT-PCR shows that the expression level of CPSF1 in sh-CPSF1 cells was significantly lower than that in sh-control cells. Protein was collected 3 days after induction with 1 μg/ml doxycycline (DOX). The Western blot showed that the protein expression of CPSF1 in sh-CPSF1 was decreased compared with the sh-control. (TIF) [file pone.0233380.s003.tif]

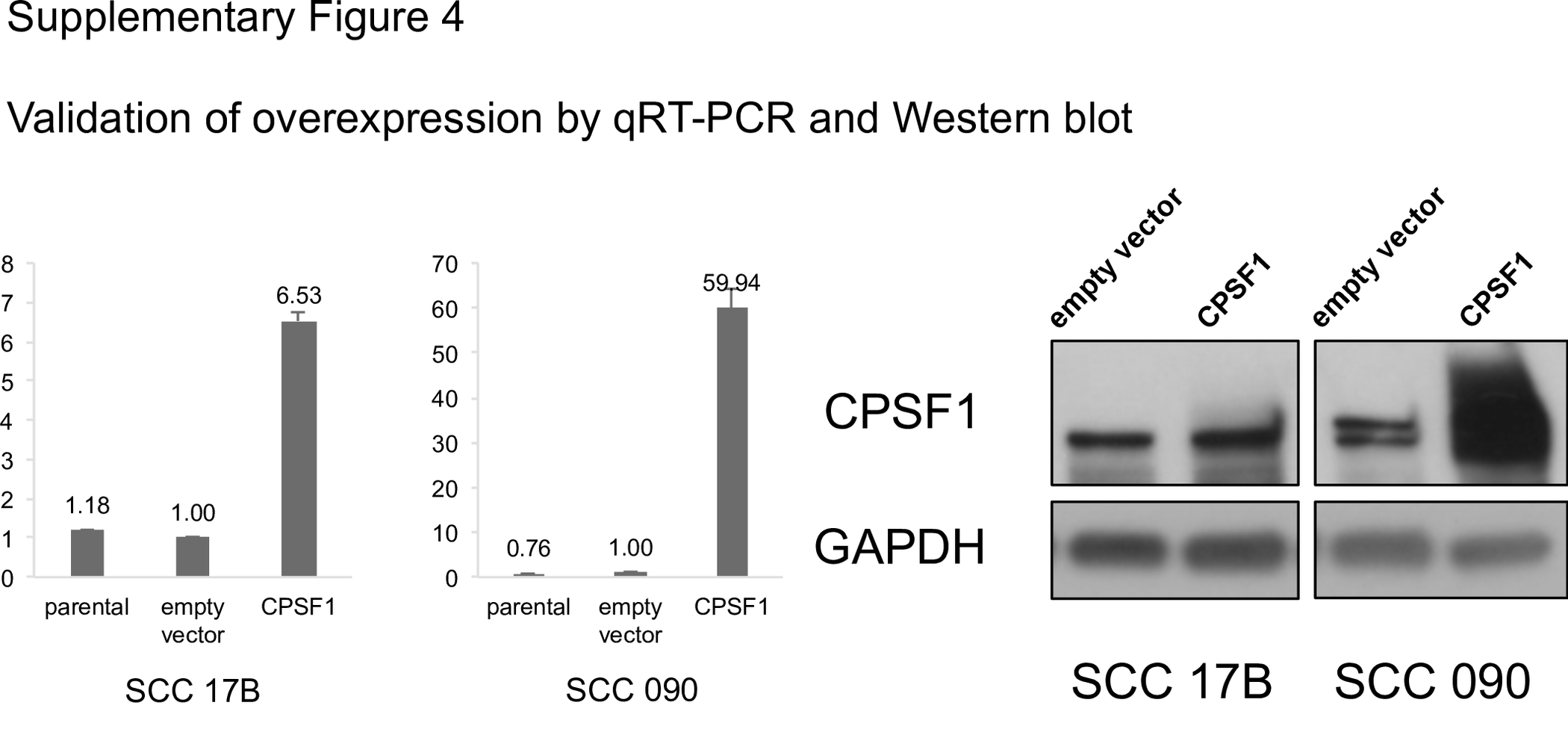

Supplement: S4 Fig — The mRNA expression level of CPSF1 in the SCC17B and SCC090 cell lines transfected with empty vector or CPSF1 overexpression vector. qRT-PCR showed that the expression of CPSF1 in SCC17B and SCC090 cell lines after transfection with CPSF1 was significantly higher than in empty vector cells. The Western blot showed that the protein expression of CPSF1 was overexpressed compared with the empty vector. (TIF) [file pone.0233380.s004.tif]

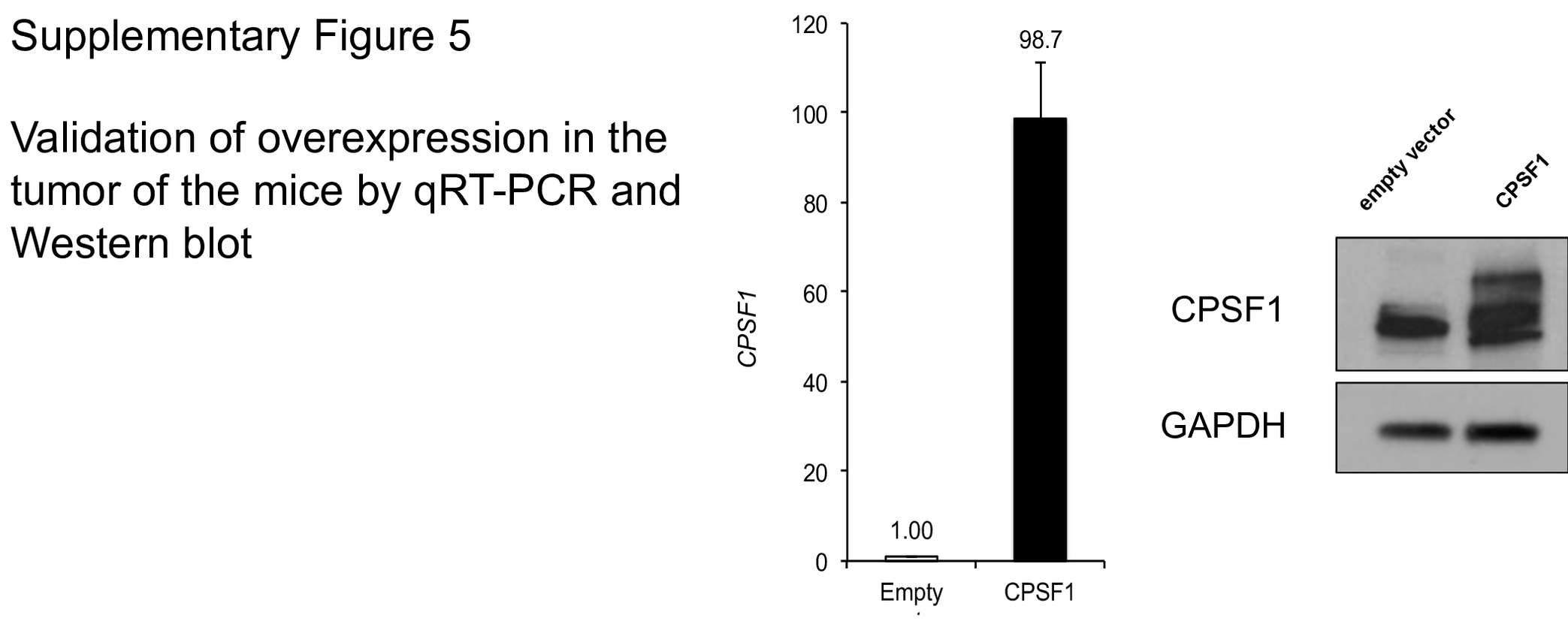

Supplement: S5 Fig — CPSF1 overexpression was validated by Western blot and qRT-PCR. CPSF1 expression in the tumor of overexpressed CPSF1 group was higher than empty vector group by qRT-PCR and Western blot. (TIF) [file pone.0233380.s005.tif]

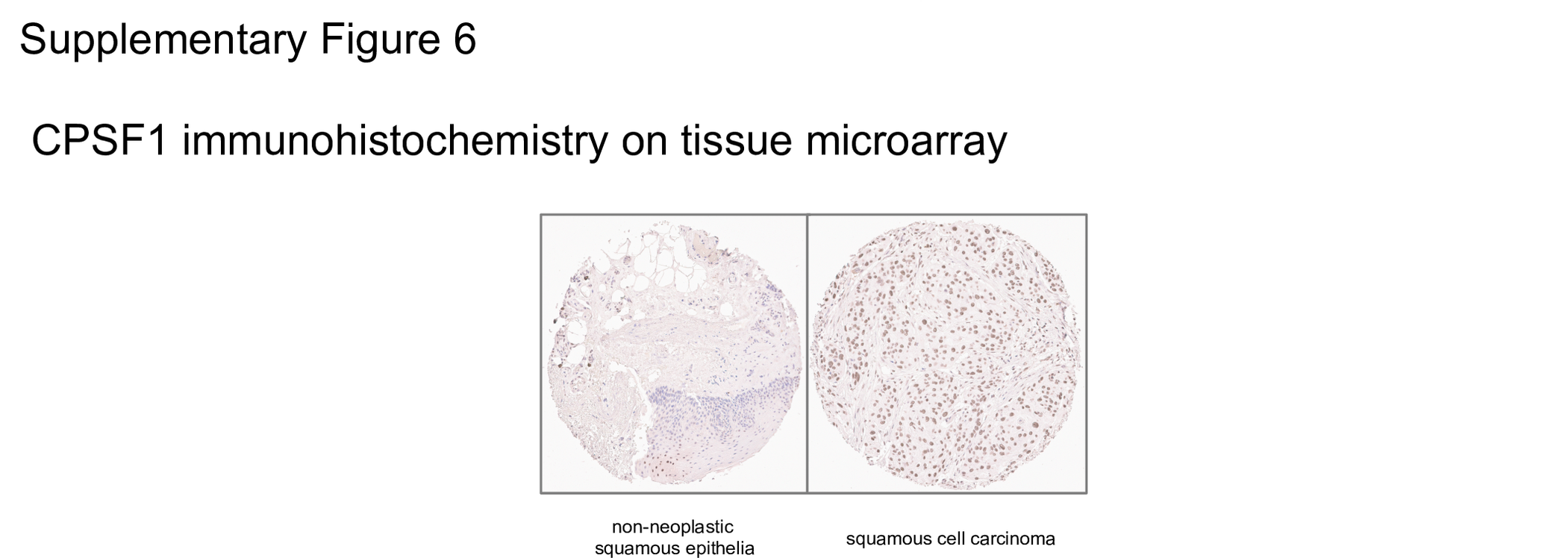

Supplement: S6 Fig — Representative non-neoplastic epithelia and squamous cell carcinoma cores from the tissue microarray with CPSF1 immunohistochemistry staining. Overexpression of CPSF1 in head and neck squamous cell carcinoma tumor was seen compared to non-neoplastic squamous epithelial tissue. (TIF) [file pone.0233380.s006.tif]

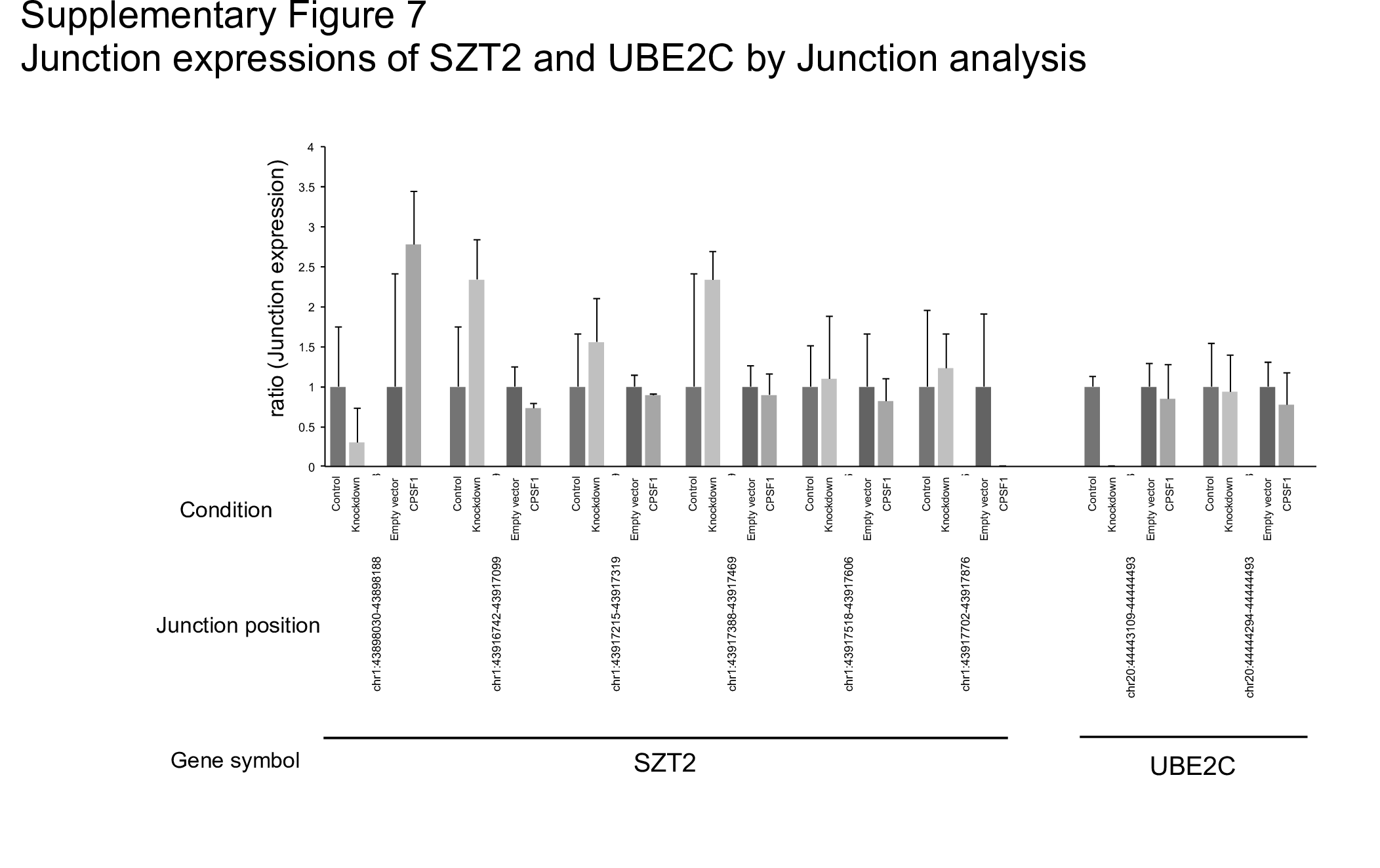

Supplement: S7 Fig — Some junction expressions in SZT2 were decreased in knockdown dataset and increased in overexpression dataset or increased in knockdown dataset and decreased in overexpression dataset. However, junction expressions in UBE2C didn’t show the reversed changes between two datasets. (TIF) [file pone.0233380.s007.tif]

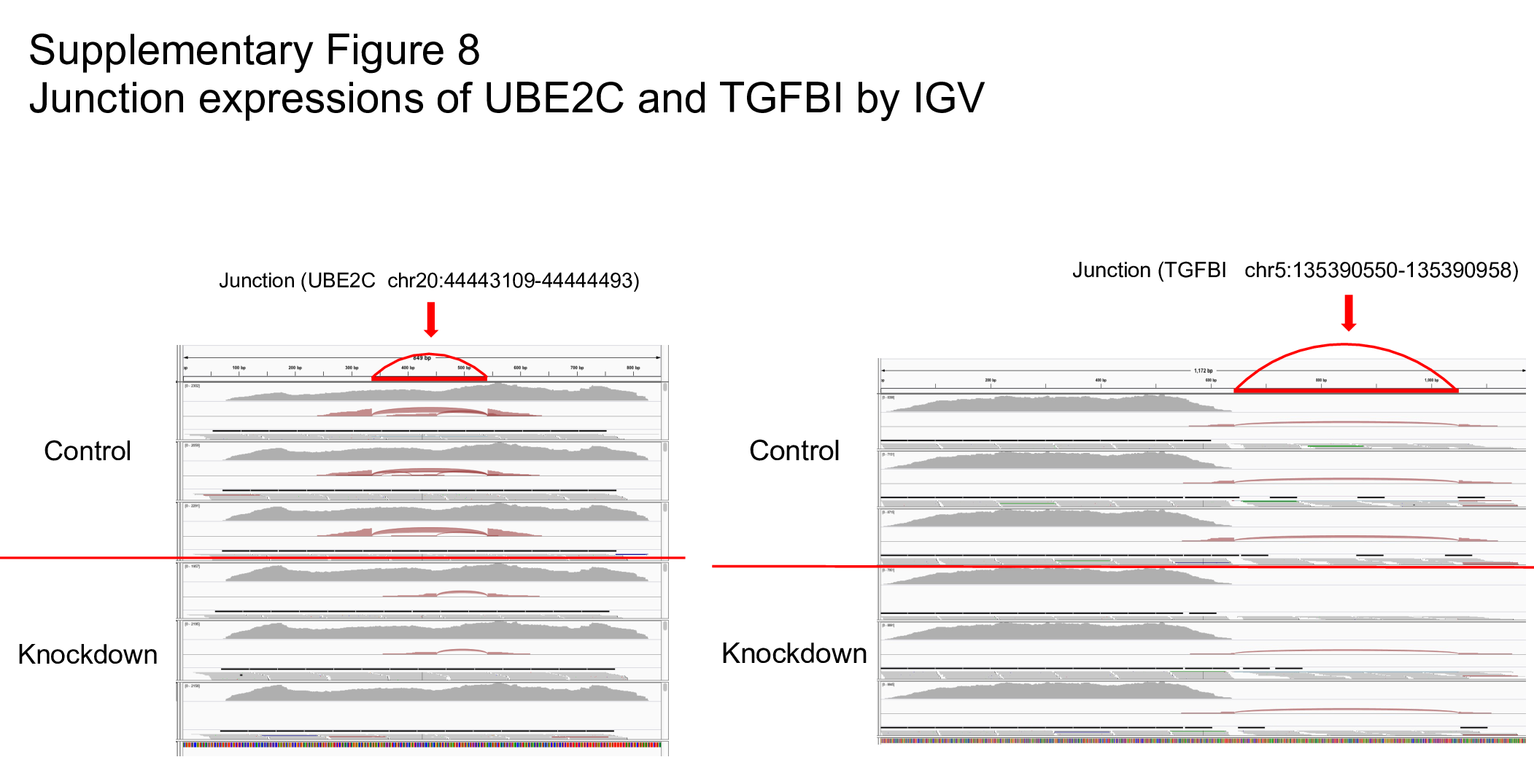

Supplement: S8 Fig — Junction expression of UBE2C (chr20:44443109–44444493) and TGFBI (chr5:135390550–135390958) were confirmed by Sashimi plot on the Integrative Genomics Viewer (IGV) using raw RNA-Seq data. Junctions were decreased by knockdown of CPSF1. (TIF) [file pone.0233380.s008.tif]

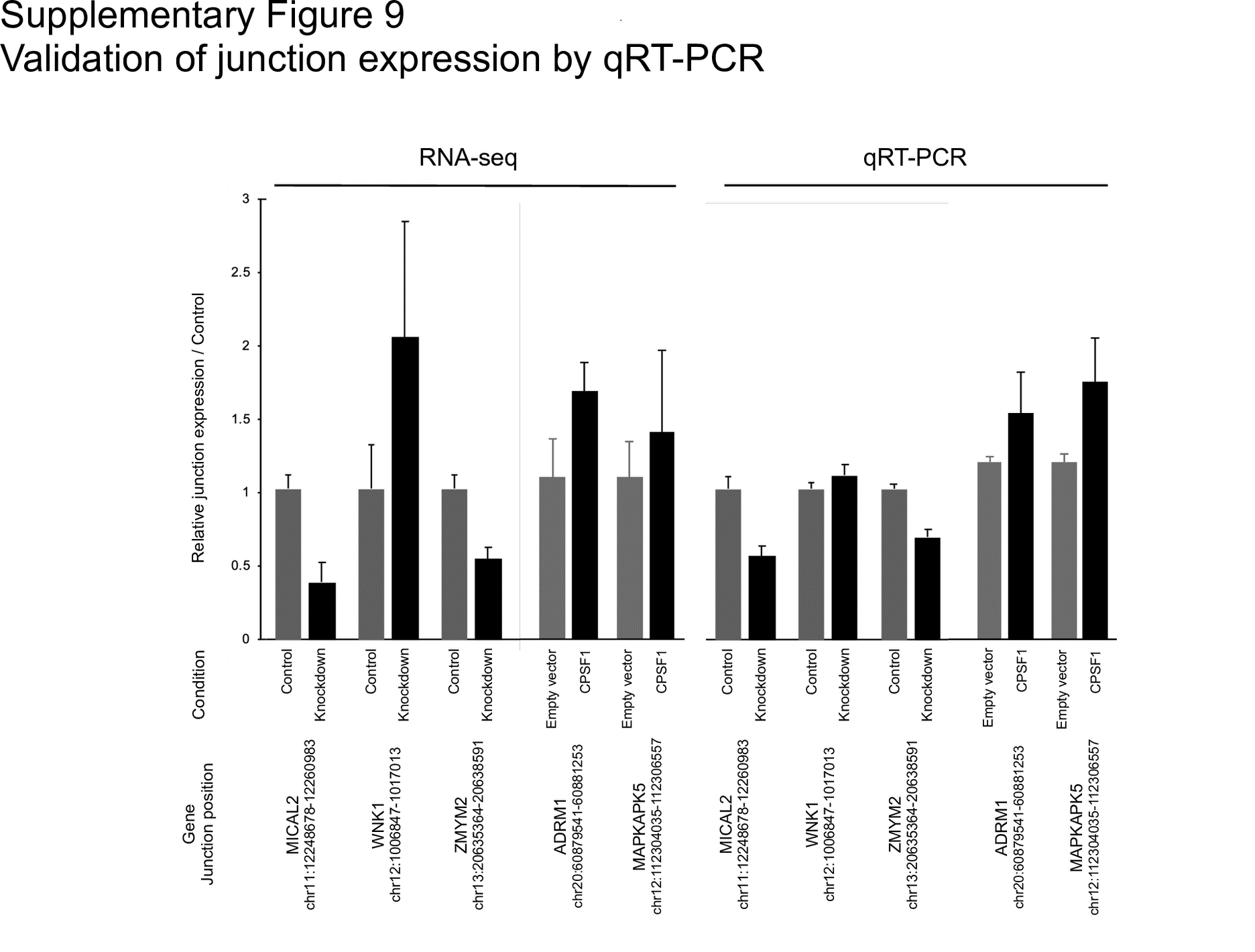

Supplement: S9 Fig — To validate the junction expression, several junctions (MICAL2 (chr11:12248678–12260983), WNK1 (chr12:1006847–1017013), ZMYM2 (chr13:20635364–20638591), ADRM1 (chr20:60879541–60881253) and MAPKAPK5 (chr12:112304035–112306557)) were selected for RT-PCR. The changes of these junction expressions were also confirmed by RT-PCR. (TIF) [file pone.0233380.s009.tif]

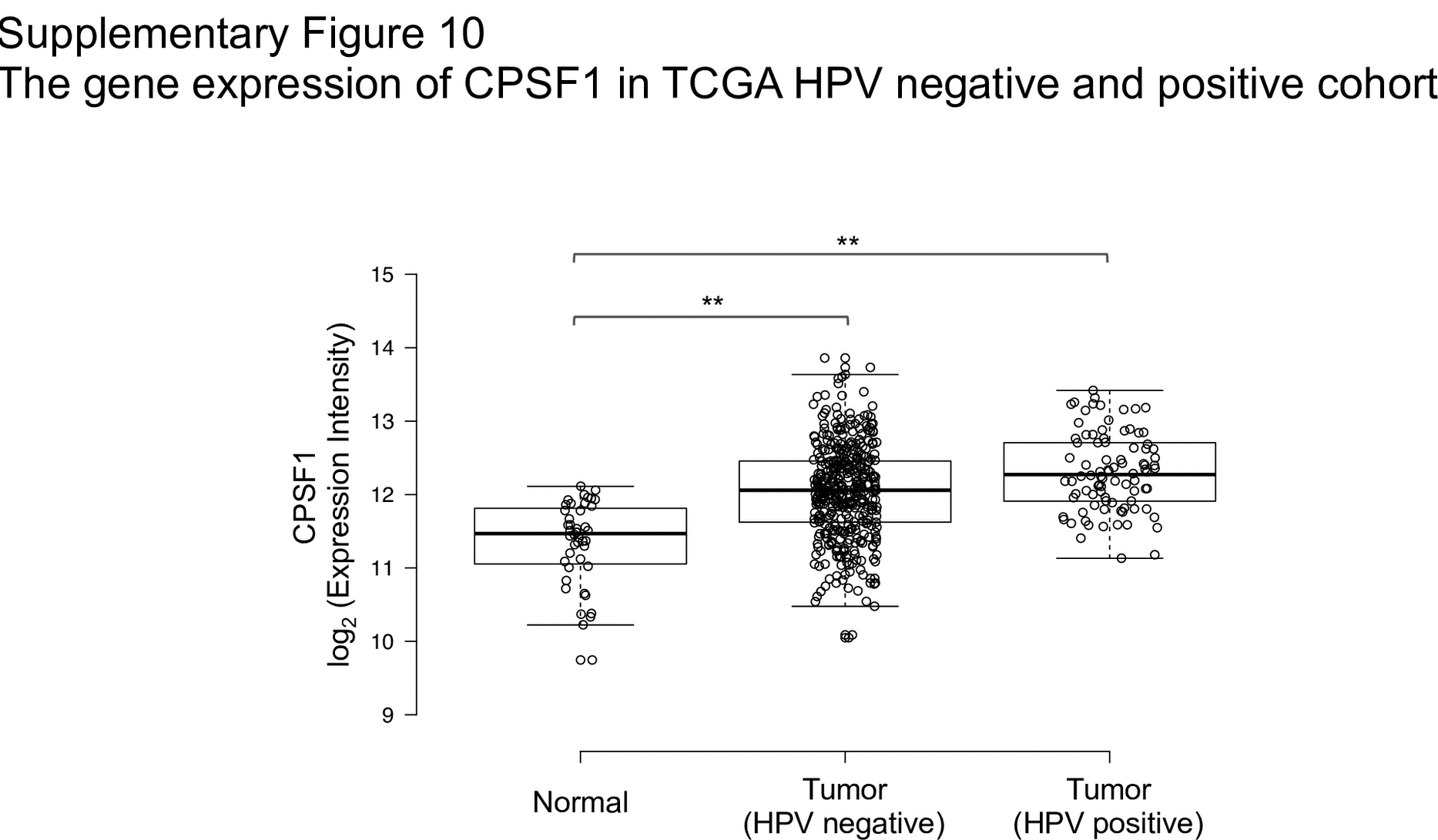

Supplement: S10 Fig — The gene expression of CPSF1 in TCGA HPV negative and positive cohort. In the comparison of CPSF1 gene expression between normal and tumor in each TCGA HPV negative and positive cohort, a significant high gene expression in tumor was confirmed. P value was calculated using Student’s t-test. **: P < 0.001. (TIF) [file pone.0233380.s010.tif]

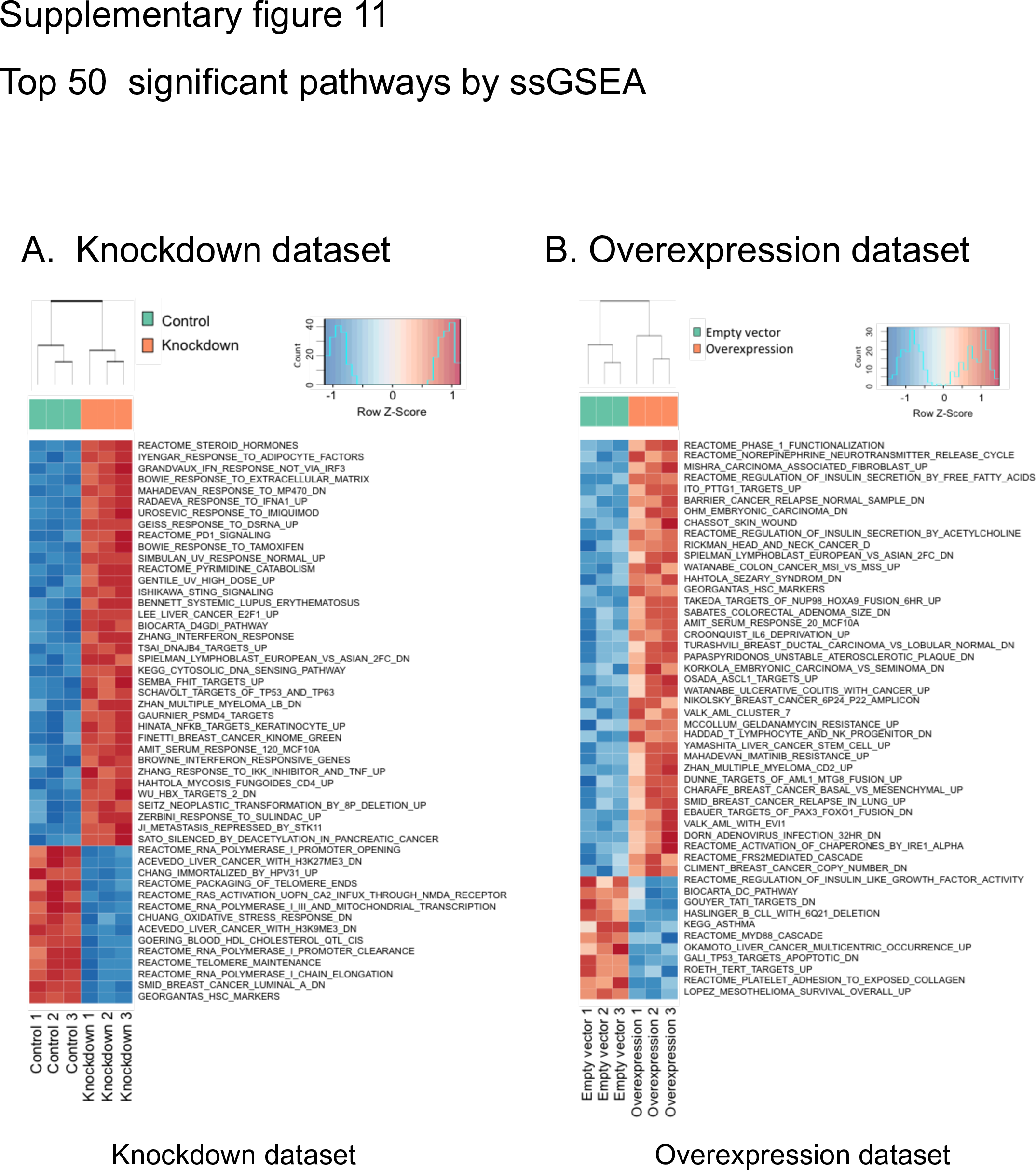

Supplement: S11 Fig — A Top 50 significant pathways of Knockdown dataset by ssGSEA. Six significant cancer-related gene sets were found in the knockdown dataset, including two well-known pathways, such as metastasis or RAS activation.B Fig. Top 50 significant pathways of Overexpression dataset by ssGSEA. Twelve cancer-associated gene sets were identified in the overexpression data set. A well-known TP53 pathway, GALI_TP53_TARGETS_APOPTOTIC_DN, was found in this dataset as well. (TIF) [file pone.0233380.s011.tif]

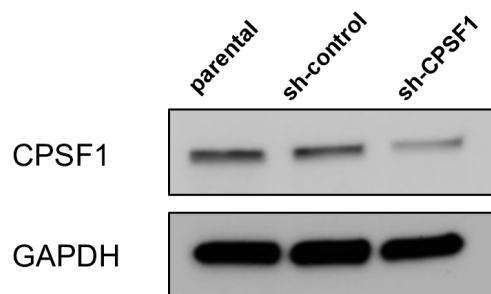

BICR22

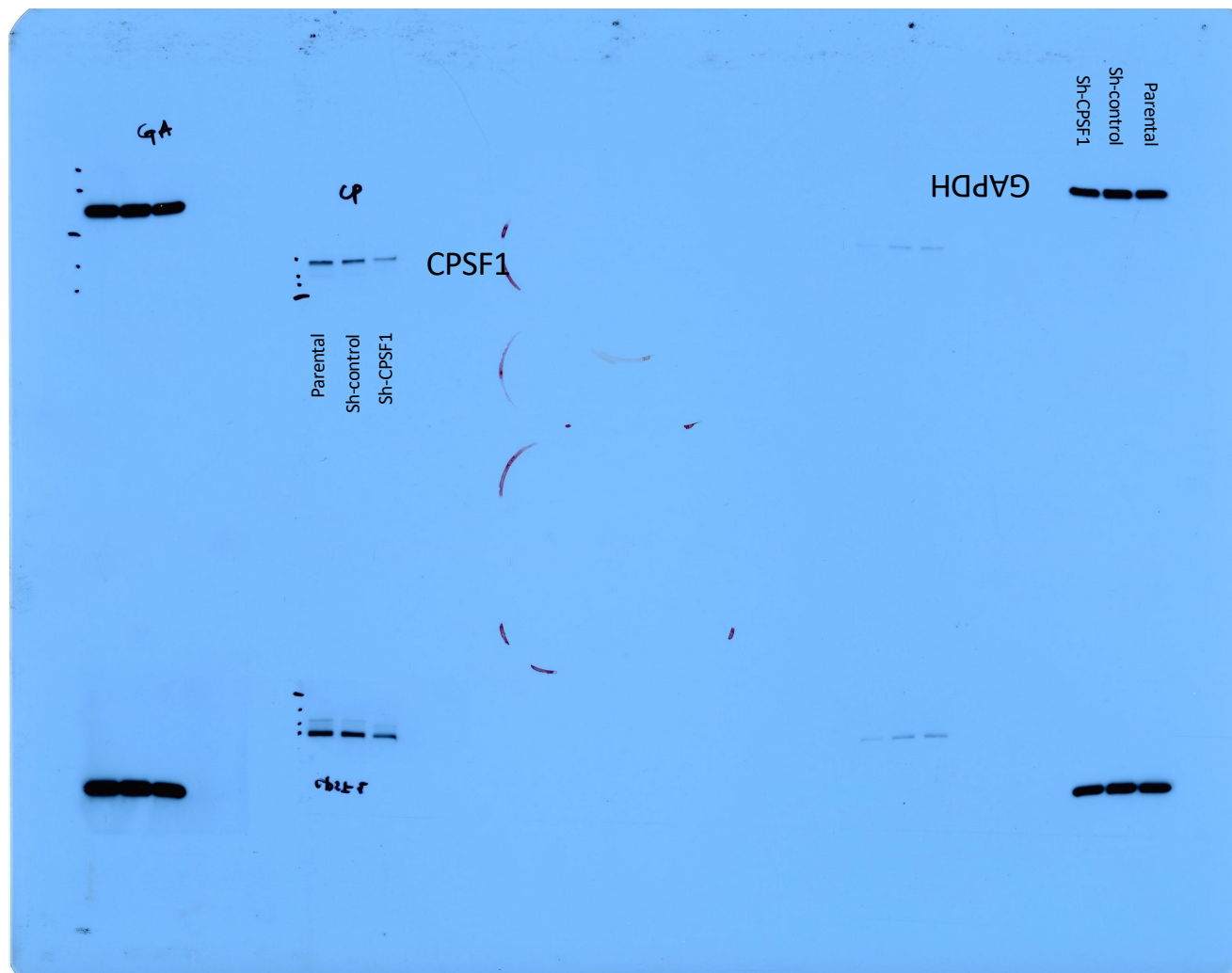

Supplement: S1 Raw images — (PDF) [file pone.0233380.s023.pdf]

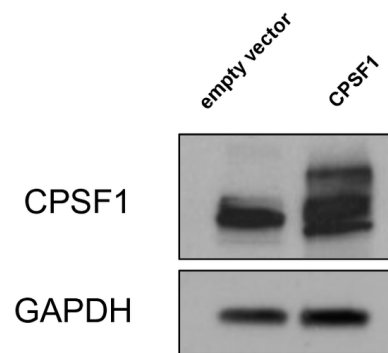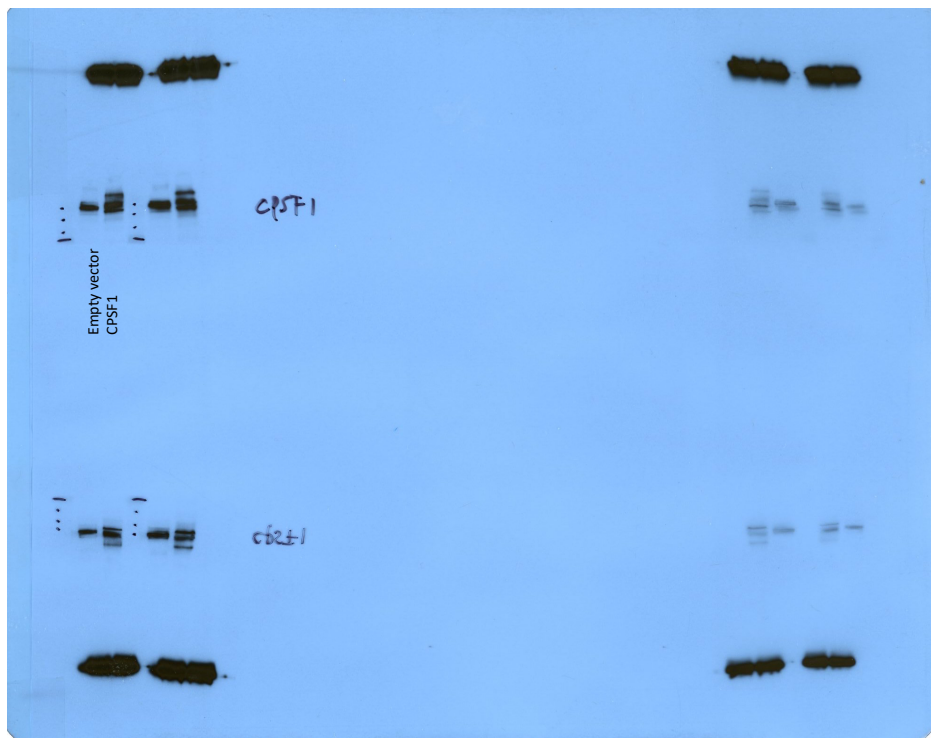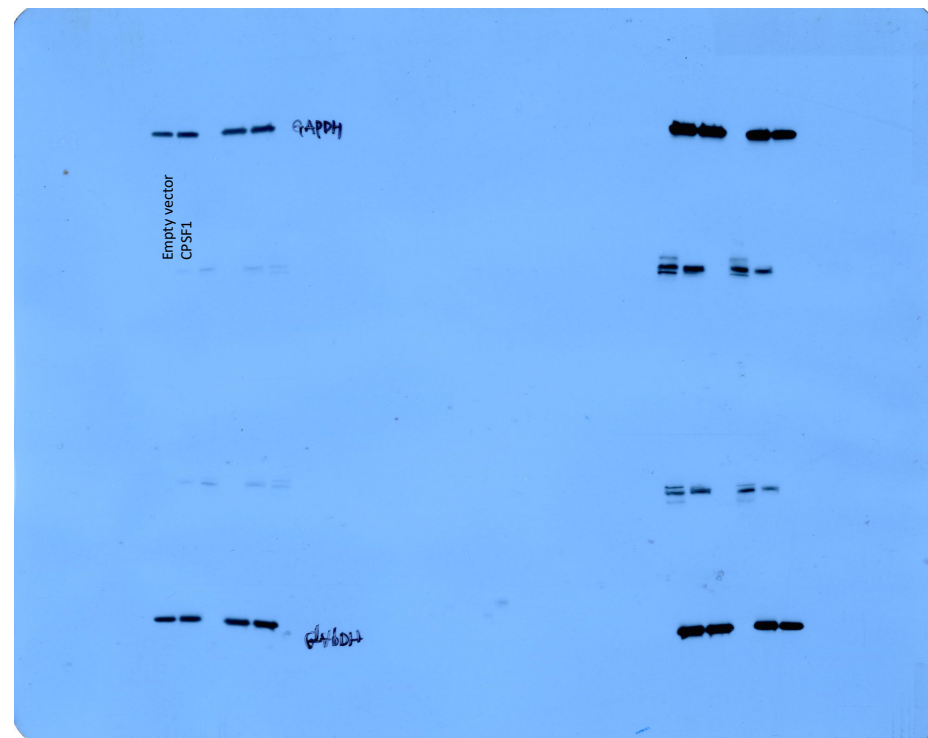

Supplement: S3 Raw images — (PDF) [file pone.0233380.s025.pdf]
