# Supplementary material for: Aberrant expression of CPSF1 promotes head and neck squamous cell carcinoma via regulating alternative splicing
Source: PLoS One. 2020 May 21;15(5):e0233380. doi: 10.1371/journal.pone.0233380 (PMC7241804; doi:10.1371/journal.pone.0233380)
Supplement: S9 Table — (PDF) [file pone.0233380.s020.pdf]

gene symbol	Gene Name	p value
PNISR	PNN interacting serine and arginine rich protein	0.000035
SLC39A1	solute carrier family 39 member 1	0.00041
LAMC2	laminin subunit gamma 2	0.00061
UBE2C	ubiquitin conjugating enzyme E2 C	0.0037
PUM2	pumilio RNA binding family member 2	0.0057
SLC5A6	solute carrier family 5 member 6	0.0091
HRAS	HRas proto-oncogene, GTPase	0.01
AKT2	AKT serine/threonine kinase 2	0.011
HDLBP	high density lipoprotein binding protein	0.011
TK1	thymidine kinase 1	0.011
TXN2	thioredoxin 2	0.011
MIR4435-1HG	MIR4435-2 host gene	0.013
EIF4G1	eukaryotic translation initiation factor 4 gamma 1	0.015
NIN	ninein	0.015
MICAL2	microtubule associated monooxygenase, calponin and LIM domain containing 2	0.017
PIGO	phosphatidylinositol glycan anchor biosynthesis class O	0.019
PRMT5	protein arginine methyltransferase 5	0.019
ZNF618	zinc finger protein 618	0.024
MIR205HG	MIR205 host gene	0.027
UBR3	ubiquitin protein ligase E3 component n-recognin 3	0.028
PSME4	proteasome activator subunit 4	0.029
SETX	senataxin	0.029
TGFBI	transforming growth factor beta induced	0.03
ZMYM2	zinc finger MYM-type containing 2	0.033
IFI16	interferon gamma inducible protein 16	0.036
SMEK2(PPP4R3B)	Protein Phosphatase 4 Regulatory Subunit 3B	0.037
ERO1L	endoplasmic reticulum oxidoreductase 1 alpha	0.038
GPX1	glutathione peroxidase 1	0.038
WNK1	WNK lysine deficient protein kinase 1	0.038
SZT2	seizure threshold 2 homolog	0.04
PRDX5	peroxiredoxin 5	0.042
PHF15(JADE2)	Jade Family PHD Finger 2(JADE2)	0.044
SLC38A1	solute carrier family 38 member 1	0.044
RP11-104H15.10		0.047
PTK7	protein tyrosine kinase 7	0.048
TMEM147	transmembrane protein 147	0.048
RPL17	ribosomal protein L17	0.049
TAF10	TATA-box binding protein associated factor 10	0.049
VMP1	vacuole membrane protein 1	0.049

Supplementary Table 9

The list of significant genes after junction analysis of the knockdown dataset
