# Supplementary material for: Aberrant expression of CPSF1 promotes head and neck squamous cell carcinoma via regulating alternative splicing
Source: PLoS One. 2020 May 21;15(5):e0233380. doi: 10.1371/journal.pone.0233380 (PMC7241804; doi:10.1371/journal.pone.0233380)
Supplement: S10 Table — (PDF) [file pone.0233380.s021.pdf]

Gene symbol	Gene name	p.value
BOK	BOK, BCL2 Family Apoptosis Regulator	0.00065
MAP4	Microtubule Associated Protein 4	0.00065
ATG13	Autophagy Related 13	0.0018
FANCD2	Fanconi anemia, complementation group D2	0.0019
AKNAD1	AKNA Domain Containing 1	0.0023
SZT2	SZT2, KICSTOR Complex Subunit	0.0035
MB21D1	Mab-21 Domain Containing 1	0.0046
BPNT1	3'(2'), 5'-Bisphosphate Nucleotidase 1	0.0059
CTC-432M15.3	ENSG00000273217 Gene	0.0061
SLC25A19	Solute Carrier Family 25 Member 19	0.0065
AP1G1	Adaptor Related Protein Complex 1 Gamma 1 Subunit	0.0068
POLR3E	RNA Polymerase III Subunit B	0.009
ZDHHC23	Zinc Finger DHHC-Type Containing 23	0.0093
RUVBL1	RuvB-like 1 (E. coli)	0.0094
MTUS1	Microtubule Associated Scaffold Protein 1	0.01
TRIB3	Tribbles Pseudokinase 3	0.013
ACSL5	Acyl-CoA Synthetase Long-Chain Family Member 5	0.014
PYGO2	Pygopus Family PHD Finger 2	0.014
ELAVL1	ELAV Like RNA Binding Protein 1	0.015
NEK9	NIMA (never in mitosis gene a)- related kinase 9	0.015
RPS14	Ribosomal Protein S14	0.015
AC092755.4	ENSG00000227161 Gene	0.016
ADRM1	Adhesion Regulating Molecule 1	0.016
HNRNPH1	Heterogeneous Nuclear Ribonucleoprotein H1	0.017
PLAA	Phospholipase A2 Activating Protein	0.018
ENTPD1-AS1	ENTPD1 Antisense RNA 1	0.019
CSPG4P12	Chondroitin Sulfate Proteoglycan 4 Pseudogene 12	0.021
ATPAF1	ATP synthase mitochondrial F1 complex assembly factor 1	0.022
EGLN1	Egl-9 Family Hypoxia Inducible Factor 1	0.022
CTA-293F17.1	Uncharacterized LOC101927811	0.023
HPS5	Hermansky-Pudlak syndrome 5	0.024
PSMD4	Proteasome 26S Subunit, Non-ATPase 4	0.025
RAB3IP	RAB3A interacting protein (rabin3)	0.025
SLC25A12	Solute Carrier Family 25 Member 12	0.025
PPP2R2D	Protein Phosphatase 2 Regulatory Subunit Bdelta	0.026
COX4I1	Cytochrome C Oxidase Subunit 4I1	0.027
RPL12	Ribosomal Protein L12	0.027
MAPKAPK5	Mitogen-Activated Protein Kinase-Activated Protein Kinase 5	0.028
ENOSF1	Enolase Superfamily Member 1	0.029
FAM111A	Family With Sequence Similarity 111 Member A	0.031
BRAT1	BRCA1 Associated ATM Activator 1	0.034
EHF	ETS Homologous Factor	0.034
EIF6	Eukaryotic Translation Initiation Factor 6	0.035
KRT8	Keratin 8	0.035
VAPA	VAMP Associated Protein A	0.037
PARP6	Poly(ADP-Ribose) Polymerase Family Member 6	0.038
NMRAL1	NmrA Like Redox Sensor 1	0.039
LIPH	Lipase H	0.04
LSM12	LSM12 homolog (S. cerevisiae)	0.042
UBE2C	Ubiquitin Conjugating Enzyme E2 C	0.043
RDH11	Retinol Dehydrogenase 11 (All-Trans/9-Cis/11-Cis)	0.044
NFS1	NFS1, Cysteine Desulfurase	0.045
TNC	Tenascin C	0.047
TMSB4X	Thymosin Beta 4, X-Linked	0.049
TPI1	TPI1 pseudogene; triosephosphate isomerase 1	0.049
ATP2C2	ATPase, Ca++ transporting, type 2C, member 2	0.05

Supplementary Table 10

The list of significant genes after junction analysis of the overexpression dataset
