# Supplementary material for: Aberrant expression of CPSF1 promotes head and neck squamous cell carcinoma via regulating alternative splicing
Source: PLoS One. 2020 May 21;15(5):e0233380. doi: 10.1371/journal.pone.0233380 (PMC7241804; doi:10.1371/journal.pone.0233380)
Supplement: S11 Table — (PDF) [file pone.0233380.s022.pdf]

Gene symbol	Junction	Presence of binding sites	Number of binding sites
BOK	chr2:242498408－242509540	+	9
MAP4	chr3:47917175－47917251	+	1
ATG13	chr11:46670733－46671808	+	6
FANCD2	chr3:10116357－10122784	+	13
AKNAD1	chr1:109484027－109486153	+	4
SZT2	chr1:43916742－43917099	-	0
MB21D1	chr6:74133546－74134580	+	4
BPNT1	chr1:220247309－220263112	+	30
CTC-432M15.3	chr5:131039842－131039926	+	3
SLC25A19	chr17:73269074－73279674	+	27
AP1G1	chr16:71798549－71803598	+	12
POLR3E	chr16:22320831－22321172	+	1
ZDHHC23	chr3:113667037－113667616	+	1
RUVBL1	chr3:127783621－127801417	+	3
MTUS1	chr8:17611366－17613362	+	2
TRIB3	chr20:362035－368655	+	5
ACSL5	chr10:114173065－114177604	+	7
PYGO2	chr1:154935186－154936111	+	1
ELAVL1	chr19:8023463－8038762	+	16
NEK9	chr14:75572889－75573359	+	3

Supplemental Table 11. Presence of the binding site of CPSF1 around ASE associated with CPSF1 overexpression
