# Supplementary material for: Aberrant expression of CPSF1 promotes head and neck squamous cell carcinoma via regulating alternative splicing
Source: PLoS One. 2020 May 21;15(5):e0233380. doi: 10.1371/journal.pone.0233380 (PMC7241804; doi:10.1371/journal.pone.0233380)
Supplement: S2 Raw images — (PDF) [file pone.0233380.s024.pdf]

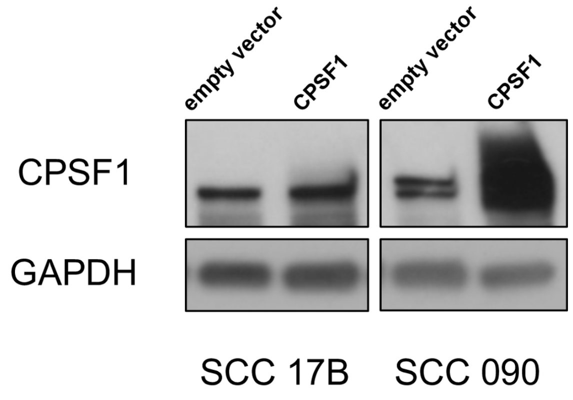
empty vector
CPSF1
empty vector
CPSF1
CPSF1
GAPDH
SCC 17B
SCC 090

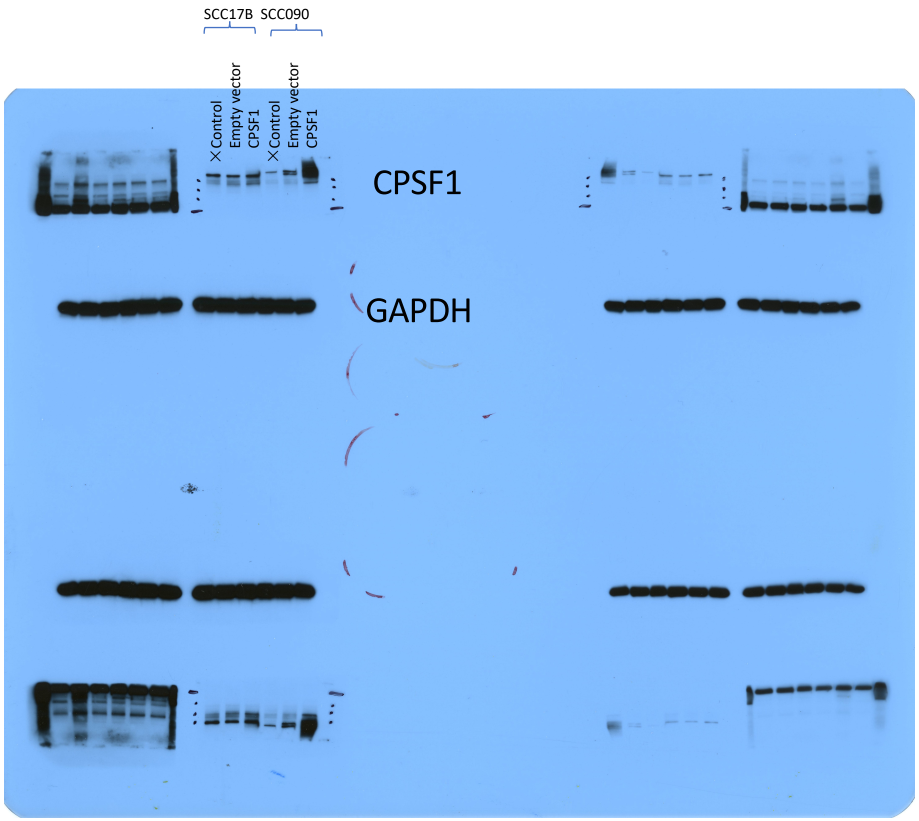
SCC17B
SCC090
Control
Empty vector
CPSF1
Control
Empty vector
CPSF1
CPSF1
GAPDH

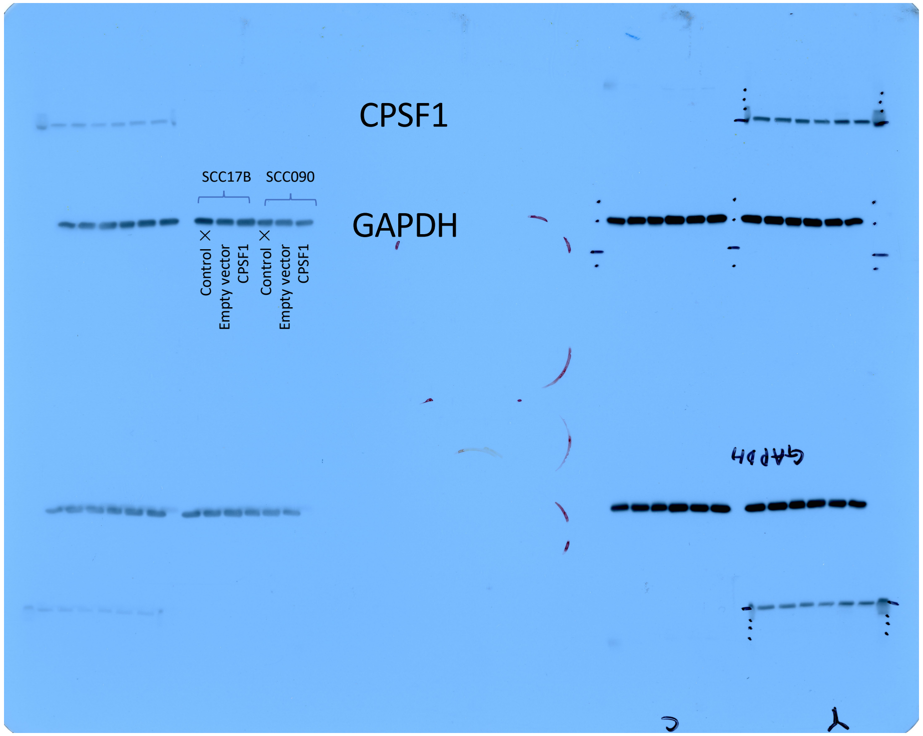
CPSF1
SCC17B
SCC090
Control
Empty vector
CPSF1
Control
Empty vector
CPSF1
GAPDH
